# VHL suppresses RAPTOR and inhibits mTORC1 signaling in clear cell renal cell carcinoma

**DOI:** 10.1038/s41598-021-94132-5

**Published:** 2021-07-21

**Authors:** Athina Ganner, Christina Gehrke, Marinella Klein, Lena Thegtmeier, Tanja Matulenski, Laura Wingendorf, Lu Wang, Felicitas Pilz, Lars Greidl, Lisa Meid, Fruzsina Kotsis, Gerd Walz, Ian J. Frew, Elke Neumann-Haefelin

**Affiliations:** 1 Renal Division, Department of Medicine, Medical Center, Faculty of Medicine, University of Freiburg, Freiburg, Germany; 2 Department of Internal Medicine I, Medical Center - University of Freiburg, Faculty of Medicine, University of Freiburg, Freiburg, Germany

**Keywords:** Renal cell carcinoma, Mechanisms of disease

## Abstract

Inactivation of the tumor suppressor von Hippel–Lindau (*VHL*) gene is a key event in hereditary and sporadic clear cell renal cell carcinomas (ccRCC). The mechanistic target of rapamycin (mTOR) signaling pathway is a fundamental regulator of cell growth and proliferation, and hyperactivation of mTOR signaling is a common finding in VHL-dependent ccRCC. Deregulation of mTOR signaling correlates with tumor progression and poor outcome in patients with ccRCC. Here, we report that the regulatory-associated protein of mTOR (RAPTOR) is strikingly repressed by VHL. VHL interacts with RAPTOR and increases RAPTOR degradation by ubiquitination, thereby inhibiting mTORC1 signaling. Consistent with hyperactivation of mTORC1 signaling in VHL-deficient ccRCC, we observed that loss of *vhl-1* function in *C. elegans* increased mTORC1 activity, supporting an evolutionary conserved mechanism. Our work reveals important new mechanistic insight into deregulation of mTORC1 signaling in ccRCC and links VHL directly to the control of RAPTOR/mTORC1. This may represent a novel mechanism whereby loss of VHL affects organ integrity and tumor behavior.

## Introduction

Renal cell carcinoma (RCC) causes more than 140,000 deaths per year worldwide^1^. RCC have several histologic subtypes, with the most common one (> 80%) being clear cell RCC (ccRCC). Bi-allelic inactivation of the *von Hippel Lindau* (*VHL*) gene is a hallmark event that arises in the majority of sporadic ccRCC cases implicating the *VHL* gene as the most important renal tumor suppressor gene in general^2,3^. Mutations of *VHL* are also causative for the inherited autosomal dominant von Hippel–Lindau syndrome manifested by a variety of benign and malignant tumors including ccRCC.

The VHL protein functions as the substrate recognition subunit of an E3 ubiquitin ligase complex targeting the hypoxia inducible transcription factor (HIF) α subunits for proteasomal degradation under normal oxygen levels. Genetic *VHL* inactivation leads to constitutive HIFα accumulation and consequently formation of HIF heterodimers, which induce transcription programs promoting bioenergetic adaptation to hypoxia. Reprogramming of numerous cellular systems by HIF contributes to the pathogenesis of ccRCC by altering metabolism, angiogenesis, extracellular matrix, invasion and apoptosis-resistance. Accumulating evidence indicates that *VHL* inactivation is not sufficient to cause renal tumor formation. Additional genetic events and cellular alterations are required as second hits for malignant transformation. Comprehensive genomic analyses of ccRCC identified epigenetic control and PI3K-mechanistic Target of Rapamycin (mTOR) pathways as major determinants in ccRCC pathogenesis^4,5^.

In ccRCC the mTOR pathway is commonly hyperactivated^6,7^. Dysregulation of mTORC1 signaling plays a key role in the oncogenesis and progression of ccRCC, and hyperactivation of mTOR correlates with poor outcome in ccRCC patients. Hence, mTOR inhibitors (such as everolimus and temsirolimus) have been approved for treatment of advanced RCCs, but therapy resistance develops in most patients^8^.

The mTOR kinase is a highly conserved and fundamental regulator of cell growth, metabolism, and proliferation in all eukaryotes^9^. mTOR constitutes the catalytic subunit of two distinct complexes, mTORC1 and mTORC2. mTORC1 contains three core components: mTOR, mLST8 and its unique defining subunit, the regulatory-assoiated protein of mTOR (RAPTOR). Diverse extra- and intra-cellular signals activate mTORC1, including nutrient availability and growth factors, while hypoxia and low cellular energy levels inhibit mTORC1 activity. The primary role of mTORC1 is to initiate biosynthesis cascades for proteins, lipids and nucleotides to support cell growth, while also suppressing catabolic pathways like autophagy^10^.

The mechanisms underlying increased mTOR signaling activity in ccRCC have remained unclear. Genomic studies have found that ~ 26% of ccRCC harbor mutations in a number of PI3K-AKT-mTORC1 pathway genes^2,5^. Genetic alterations thus likely contribute to mTORC1 activation in ccRCC. Further integrated molecular studies of ccRCC revealed high levels of AKT-mTORC1 signaling also without an associated genetic alteration^11^ suggesting that additional molecular players and upstream signals are involved. Notably, VHL was recently found to directly suppress AKT activity, and in *VHL*-deficient cells AKT was activated promoting cell survival and tumorigenesis^12^. HIF is generally thought to inhibit mTORC1 in hypoxia by activating the expression of its downstream target gene *REDD1*, which subsequently activates the mTORC1 repressor tuberous sclerosis complex^13^. However, mTOR can evade inhibition by REDD1^14^. On the other hand HIF2α increases mTORC1 activity under low amino acid availability by increasing the expression of the SLC7A5 amino acid carrier^15^. It was also shown that suppression of the mTOR inhibitor DEPTOR in *VHL*-deficient ccRCC accelerated tumor cell proliferation^16^.

This study reveals new mechanistic insights into deregulation of mTORC1 in ccRCC. Combining cellular models for ccRCC and the *C.* *elegans* system we identify an additional layer of interaction of VHL and the PI3K-mTORC1 pathway and directly link VHL to control of RAPTOR, the essential scaffolding protein of mTORC1.

## Results

### VHL interacts with the mTORC1 subunit RAPTOR

**Figure 1:**
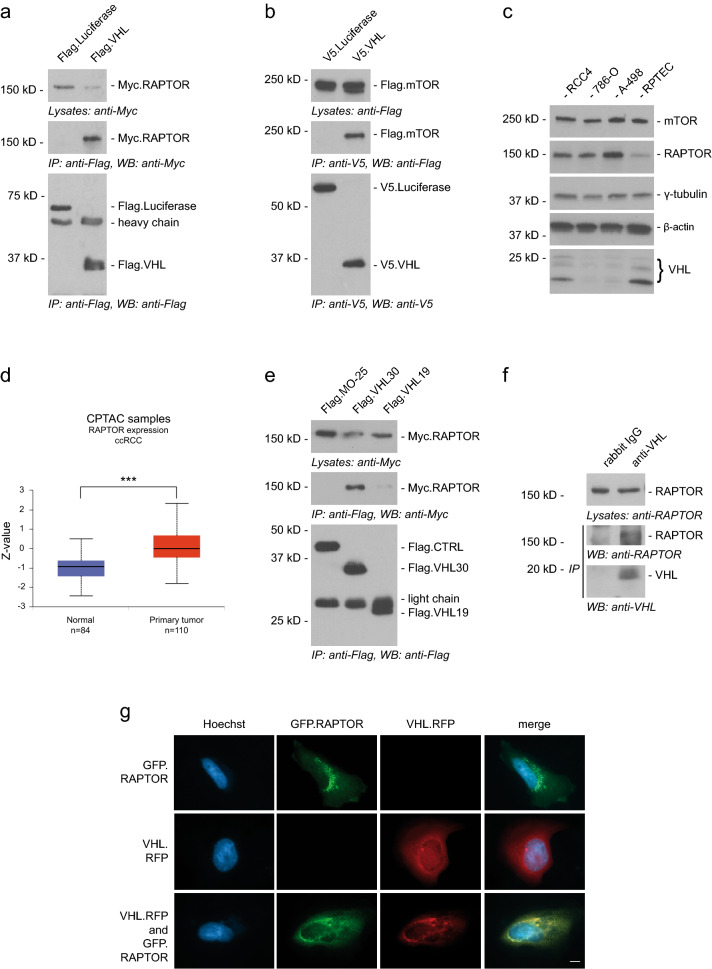
VHL interacts and co-localizes with RAPTOR. (**a**) VHL binds RAPTOR. HEK293T cells were co-transfected with Flag.VHL and Myc.RAPTOR, or Flag.Luciferase as control. Cell lysates were used for detecting RAPTOR expression by anti-Myc antibody and for immunoprecipitation (IP) by anti-Flag antibody. The lower panel shows the IP of Flag-tagged proteins. Co-immunoprecipitation of RAPTOR was detected by anti-Myc (middle panel). kD, kilodalton. Full-length blots are presented in Supplementary Fig. 4. (**b**) VHL interacts with mTOR. Flag.mTOR and V5.VHL were transiently co-expressed in HEK293T cells. V5.Luciferase was used as control. After immunoprecipitation (IP) with anti-V5 antibody, the immobilized mTOR was detected by Western blot (WB) analysis using anti-Flag antibody in the precipitate containing VHL, but not Luciferase (middle panel). Full-length blots are presented in Supplementary Fig. 4. (**c**) RAPTOR expression in upregulated in *VHL*-deficient ccRCC cell lines. Cell lysates of RCC4, 786–O, A-498 and RPTEC cells were analyzed by Western blot using anti-RAPTOR and anti-mTOR antibodies. Equal concentrations of total protein were determined by Bradford assay. β-actin and γ-tubulin levels were used as a loading control. Full-length blots are presented in Supplementary Fig. 4. (**d**) RAPTOR expression data from the UALCAN database comparing ccRCC to normal tissue, ***p ≤ 0.001. (**e**) VHL30 but not VHL19 interacts with RAPTOR. Myc.RAPTOR and Flag.VHL30 or Flag.VHL19 were transiently co-expressed in HEK293T cells. Flag.MO-25 was used as control. After immunoprecipitation (IP) with anti-Flag antibody, the immobilized RAPTOR was detected by Western blot (WB) analysis using anti-Myc antibody in the precipitate containing VHL30, but not VHL19 (middle panel). Full-length blots are presented in Supplementary Fig. 4. (**f**) Endogenous RAPTOR interacts with VHL. Endogenous VHL was immunoprecipitated (IP) from HeLa cell lysates by anti-VHL antibody (lower panel). Endogenous RAPTOR co-precipitating with VHL was detected by Western blot analysis using anti-RAPTOR antibody (middle panel). Full-length blots are presented in Supplementary Fig. 4. (**g**) Co-localization of VHL and RAPTOR. HeLa cells were transfected with GFP-tagged RAPTOR (green, upper panel) and RFP-tagged VHL (red, middle panel). Co-expression of VHL and RAPTOR revealed co-localization in peri-nuclear foci (lower panel). Fluorescent confocal microscopy images of HeLa cells. Hoechst (*blue*) was used for nuclei staining. Scale bar 10 µm.

mTORC1 is frequently hyperactivated in ccRCC and accelerates tumor progression. mTORC1 consists of the three core components mTOR, RAPTOR, and LST8. To test whether VHL can physically interact with one or more of mTORC1 core proteins, HEK293T cells were co-transfected with tagged expression constructs of VHL, RAPTOR, mTOR, and LST8 respectively. Immunoblotting analysis showed association of VHL with RAPTOR, mTOR (Fig. 1a,b), and LST8 (Supplementary Fig. 1a). Binding of VHL to the inhibitory subunits DEPTOR (DEP domain-containing mTOR-interacting protein) and PRAS40 (proline-rich Akt substrate of 40 kDa) was not observed (Supplementary. Figure 1b). As we noticed constant reduction of RAPTOR in the presence of VHL in our cell lysates by immunoblotting, we evaluated RAPTOR protein levels in different ccRCC cell lines. Interestingly, RAPTOR expression was increased in *VHL*-deficient ccRCC cell lines compared to human renal proximal tubular epithelial cells (RPTECs) (Fig. 1c). Consistent with these observations, bioinformatic analysis using the online web portal UALCAN (http://ualcan.path.uab.edu) revealed a significant upregulation of RAPTOR protein in tumor tissues of *VHL-*dependent ccRCCs when compared to normal tissues (Fig. 1d) according to the CPTAC mass-spectrometry-based proteomic tumor dataset^17^. These findings led us to investigate the interaction between VHL and RAPTOR in more detail.

The *VHL* gene encodes two biologically active isoforms, full length VHL consisting of 213 amino acid residues with a molecular mass of 30 kDa (VHL30) and an internally translated form corresponding to amino acid residues 54–213 with a molecular mass of 19 kDa (VHL19) (Supplementary Fig. 1c)^18^. Both isoforms act similarly to promote HIFα degradation and so appear to retain tumor suppressor activity, yet have isoform-specific functions^19,20^. HEK293T cells were transiently transfected with Myc-tagged RAPTOR and Flag-tagged VHL30 or VHL19. Following immunoprecipitation with Flag-antibody, immunoblotting analysis revealed that the full length VHL30 efficiently immunoprecipitates RAPTOR, while the shorter VHL19 is able to immunoprecipitate only a very small amount of RAPTOR protein, despite being expressed at similar levels to VHL30 (Fig. 1e). A similar experiment was conducted with the N-terminal fragment of VHL consisting of amino acid residues 1–53 (VHL(AA1-53)). Again, immunoprecipitation of VHL30 led to co-precipitation of RAPTOR, while VHL(AA1-53) did not associate with RAPTOR (Supplementary Fig. 1d). Together, these findings show that the VHL30 N-terminal tail, although necessary, is not sufficient for RAPTOR binding. To further confirm the interaction between VHL and RAPTOR we tested the association of endogenous proteins. HeLa cell extracts were subjected to immunoprecipitation with anti-VHL antibodies and immunoblotting analysis with a RAPTOR-specific antibody revealed the binding of endogenous RAPTOR to VHL (Fig. 1f). RAPTOR accumulates in small granules in the cytoplasm that have previously been identified as Golgi and the endoplasmic reticulum (ER)^21,22^ (Fig. 1g). VHL is predominantly cytoplasmic and can shuttle to the nucleus. In the cytoplasm, VHL proteins were shown to display ER localization^23^. Co-transfection of VHL and RAPTOR revealed a partial co-localization in cytoplasmic foci near the nucleus potentially relating to the ER (Fig. 1g).

### VHL regulates RAPTOR protein abundance

**Figure 2:**
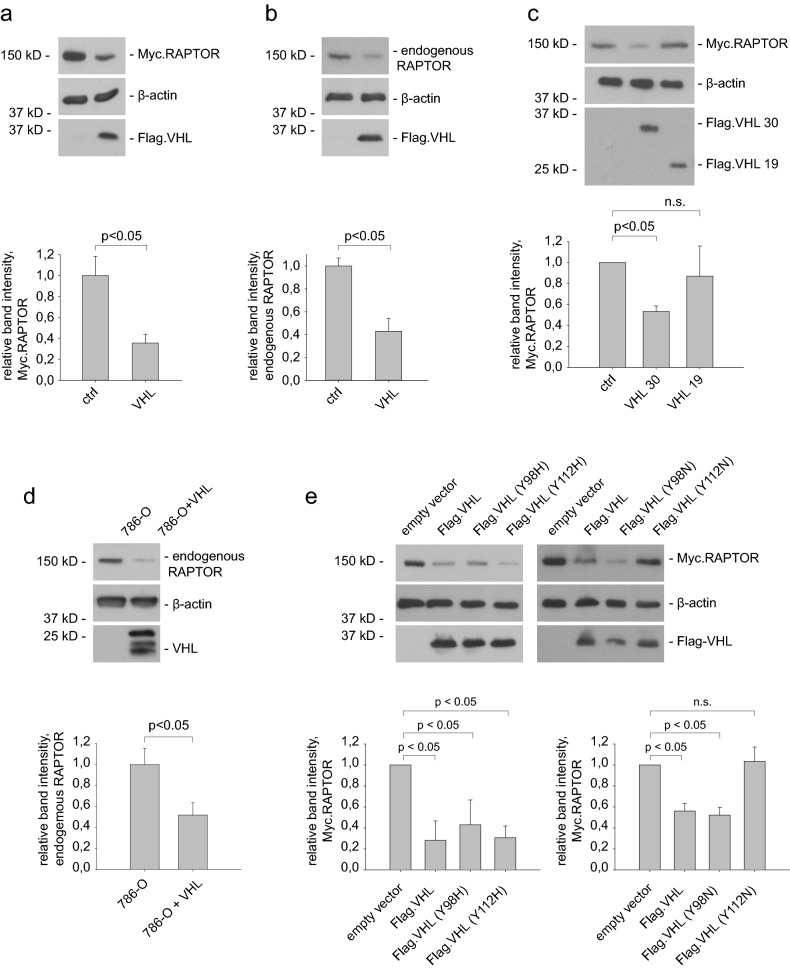
VHL regulates RAPTOR protein abundance. (**a**) VHL reduced transiently expressed RAPTOR protein. HEK293T cells were co-transfected with Myc.RAPTOR and Flag.VHL. Cell lysates were analyzed by immunoblotting using Myc- and Flag-specific antibodies. Protein levels of RAPTOR were quantified using LabImage 1D software and normalized to β-actin protein levels. The lower panel shows quantification of relative RAPTOR protein levels from three independent experiments. Data are presented as mean ± SEM. *p* < 0.05 by *t* test. kD, kilodalton. Full-length blots are presented in Supplementary Fig. 4. (**b**) VHL diminished endogenous RAPTOR levels. HEK293T cells were transiently co-transfected with Flag.VHL and serum starved overnight. Endogenous RAPTOR was detected by a specific anti-RAPTOR antibody. Lower panel shows the quantification of RAPTOR levels from three independent experiments, *p* < 0.05 (*t* test). Full-length blots are presented in Supplementary Fig. 4. (**c**) Full length VHL30 but not VHL19 regulates RAPTOR protein abundance. Immunoblotting analysis of HEK293T cells co-transfected with expression vectors for RAPTOR and control protein (Luciferase), VHL30 or VHL19. Lower panel shows the quantification of relative RAPTOR levels from three independent experiments, *p* < 0.05; n.s., not significant (*t* test). Full-length blots are presented in Supplementary Fig. 4. (**d**) RAPTOR is downregulated in VHL*-*reintroduced renal carcinoma cells. Cell lysates were prepared from *VHL*-deficient 786-O ccRCC cells and cells transduced with VHL (786-O + VHL) and assayed by immunoblotting after overnight serum withdrawal. Lower panel shows quantification of relative RAPTOR levels from three independent experiments. *p* < 0.05 (*t* test). Full-length blots are presented in Supplementary Fig. 4. (**e**) Differential regulation of RAPTOR by type 2A and 2B VHL mutants. HEK293T cells were co-transfected with expression vectors for RAPTOR and VHL wt, VHL(Y98H), VHL(112H), VHL(Y98N), and VHL(Y112N), respectively. Lower panel shows the quantification of relative RAPTOR levels from three independent experiments, *p* < 0.05; n.s., not significant (*t* test). Full-length blots are presented in Supplementary Fig. 4.

Our experiments so far suggest that VHL may have an impact on mTORC1 and most notably RAPTOR. Following this hypothesis we analyzed whether VHL alters RAPTOR protein levels under different cellular conditions. As observed before, overexpressed VHL significantly reduced the levels of RAPTOR in HEK293T cells (Fig. 2a). To confirm the effect of VHL on RAPTOR protein abundance endogenous RAPTOR protein levels were also measured. Overexpression of VHL resulted in reduced levels of endogenous RAPTOR (Fig. 2b). Since only full length VHL30 but not the short form VHL19 interacted with RAPTOR, we tested whether the VHL protein isoforms differ in their impact on RAPTOR protein abundance. Consistently, VHL30 markedly diminished RAPTOR protein levels, while VHL19 only had a weak effect (Fig. 2c). Given the prominent regulation of RAPTOR by VHL, RAPTOR protein levels in 786-O ccRCC cells were investigated. 786-O cells are a well-established cell model for ccRCC and have been previously used to show that mTOR activation drives tumorigenesis in ccRCC^7^. We re-introduced a retrovirus encoding full length VHL cDNA (VHL30) and observed downregulation of RAPTOR protein abundance as compared with *VHL-*deficient 786-O tumor cells (Fig. 2d). Together, these observations indicate that VHL suppresses RAPTOR protein abundance.

The risk for developing ccRCC seems to be linked to *VHL* genotype: type 1 mutations (large deletions and premature stops) and type 2B missense mutations are associated with a high risk, while type 2A missense mutations have lower risk^24^. To understand the basis for the suppression of RAPTOR by VHL we tested the ability of the most frequent VHL type 2A (Y98H and Y112H) and type 2B (Y98N and Y112N) representative mutants^25^ to regulate RAPTOR. The type 2A mutations Y98 H and Y112 H retained the ability to reduce RAPTOR protein levels to the same extent as wildtype VHL (Fig. 2e). Likewise, the type 2B VHL(Y98N) mutant form still suppressed RAPTOR protein levels, whereas overexpression of VHL(Y112N) mutant failed to reduce RAPTOR abundance (Fig. 2e). Mutations in residues Y98 and Y112 have been shown to affect the interaction with the key cellular substrate HIFα^25^, but we observed similar binding of VHL type 2B mutations to RAPTOR (Supplementary Fig. 2).

### VHL impairs mTORC1 signaling

**Figure 3:**
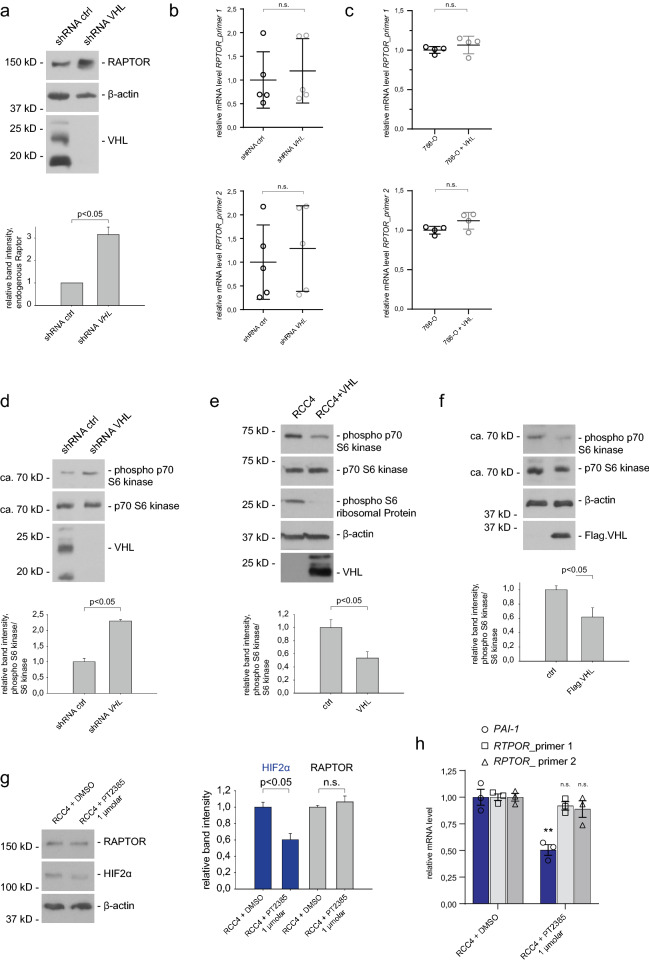
VHL modulates RAPTOR—mTORC1 signaling. (**a**) RAPTOR is upregulated in *VHL-*deficient cells. Representative blot showing RAPTOR expression in HeLa cells infected with lenti-control shRNA or lenti-*VHL* shRNA. Quantification of 3 independent experiments (lower panel). *p* < 0.05 (*t* test). Full-length blots are presented in Supplementary Fig. 4. (**b**) *RPTOR* mRNA levels are not affected by VHL. qPCR analysis of HeLa cells expressing shRNA against control or *VHL*. Two different primer sets were used for *RPTOR* mRNA analysis. n = 5 for each experiment. Data are mean ± SEM. *t* test. n.s., not significant. (**c**) Analysis of *RPTOR* mRNA levels in 786-O cells and 786-O cells expressing VHL. n = 4 for each experiment. Data are mean ± SEM. *t* test. n.s., not significant. (**d**) mTORC1 is activated in VHL-depleted cells. The levels and phosphorylation of p70 S6 kinase in HeLa shRNA cells were determined by Western blotting. Quantification of three independent experiments. *t* test; mean values ± SEM. Full-length blots are presented in Supplementary Fig. 4 (**e**) VHL inhibits phosphorylation of p70S6K and S6 ribosomal protein in RCC4 cells. Cell lysates of VHL-deficient RCC4 cells and RCC4 cells transduced with VHL were serum starved overnight and analyzed by western blot. Quantification of three independent experiments. *t* test; mean values ± SEM. Full-length blots are presented in Supplementary Fig. 4 (**f**) Overexpression of VHL reduces phosphorylation of p70 S6K. HEK293T cells were transiently transfected with Flag.VHL. Quantification of phospho-p70 S6K/ p70 S6K from three independent experiments; mean ± SEM, *p* < 0.05 (*t* test). Full-length blots are presented in Supplementary Fig. 4. (**g**) RAPTOR protein and mRNA levels are independent of HIF2α inhibition. RCC4 cells were incubated with the HIF2α inhibitor PT2385 for 72 h and lysates were assessed by immunoblotting with anti- RAPTOR and anti-HIF2α antibody. Quantification of three independent experiments. *t* test; mean values ± SEM. Full-length blots are presented in Supplementary Fig. 4. (**h**) RCC4 cells were incubated with the HIF2α inhibitor PT2385 for 72 h at concentrations indicated. mRNA expression of *RPTOR* and *PAI-1* were assessed by q-PCR relative to HSPCB. Data are represented as mean ± SEM. **p < 0.01, n.s., not significant (t test).

To further investigate the regulation of RAPTOR by VHL, HeLa cells were infected with lenti-*VHL* shRNA or lenti-control shRNA. shRNA-mediated *VHL* knockdown efficiency was verified by qPCR and, as expected, depletion of *VHL* increased HIF1α (Supplementary Fig. 3a,b). In *VHL*-depleted cells RAPTOR protein levels were strongly upregulated (Fig. 3a). We also tested whether the changes in RAPTOR levels were due to altered transcription. However, knockdown of *VHL* in HeLa cells and re-expression of VHL in 786-O cells did not substantially change *RPTOR* mRNA expression (Fig. 3b,c). These results indicate that VHL modulates RAPTOR at the post-transcriptional level.

mTORC1 directly phosphorylates and activates a set of well-characterized targets, most notably p70 S6 kinase (p70 S6K). Upon shRNA-mediated knockdown of *VHL* an increase in p70 S6K phosphorylation was observed (Fig. 3d). Immunoblotting analysis of *VHL*-deficient RCC4 cells lines confirmed the activation of mTORC1 signaling and re-expression of VHL significantly decreased phosphorylation of p70 S6K and its target ribosomal protein S6 (Fig. 3e). Consistently, overexpression of VHL in HEK293T cells significantly reduced the phosphorylation of p70 S6K (Fig. 3f). Cell proliferation rates were not affected by manipulating VHL in different cell lines (Supplementary Fig. 3c) indicating that proliferation does not account for the *VHL*-dependent RAPTOR abundance and mTORC1 activation. Taken together, this experimental evidence supports the idea that VHL associates with RAPTOR and impairs mTORC1 signaling.

To test whether the interplay between VHL and RAPTOR in our system was HIF-dependent, ccRCC cells were treated with the HIF2α isoform specific inhibitor PT2385^26^.Treatment of RCC4 and 786-O cells with PT2385 significantly reduced HIF2α activity, as evidenced by decreased expression of the target gene *PAI-1*^16,27^, but did not alter RAPTOR protein levels or mRNA expression (Fig. 3g,h, and Supplementary Fig. 3d,e). HIF1α was inhibited by IDF-11774^28^, again no significant differences in RAPTOR protein expression were observed (Supplementary Fig. 3f.). Taken together, these results point to a HIF-independent link between VHL and RAPTOR.

In ccRCC, HIF-mediated upregulation of the mTORC1 inhibitor REDD1 has been described^14^. Consistent with the literature, REDD1 levels were upregulated in *VHL*-deficient 786-O cells, but these cells still expressed RAPTOR and showed high mTORC1 activity (Supplementary Fig. 3g)^12,14^ indicating that VHL suppresses RAPTOR in a REDD1-independent manner.

### VHL-1 impairs mTORC1 signaling in C. elegans

To gain further insight into the significance of the interaction between VHL and mTORC1 signaling pathways in vivo, we used the genetically tractable model *C.* *elegans*. Core components of the VHL and mTOR signaling pathways are highly evolutionary conserved in *C.* *elegans* and this model organism has provided comprehensive insights into their fundamental functions^29–32^.

**Figure 4:**
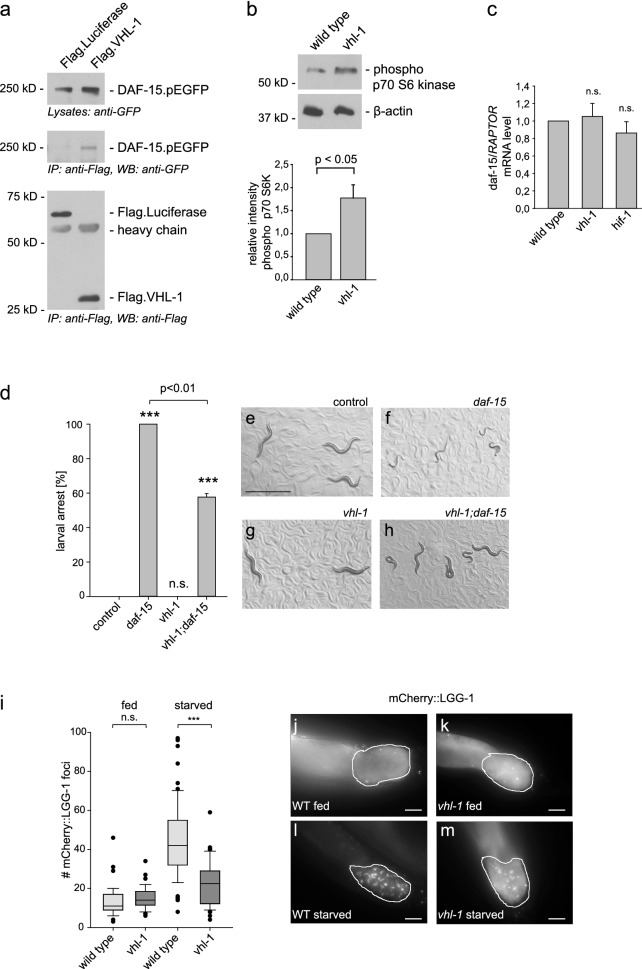
VHL-1 impairs mTORC1 signaling in *C.* *elegans.* (**a**) *C.* *elegans* VHL-1 interacts with DAF-15/RAPTOR in HEK 293 T cells. Flag-tagged VHL-1 and EGFP-tagged DAF-15 were co-expressed in HEK293T cells. After immunoprecipitation (IP) with anti-Flag antibody, the immobilized DAF-15/RAPTOR was detected by Western blot (WB) analysis using anti-GFP antibody (middle panel). The control protein Luciferase failed to bind DAF-15. Upper part shows expression of DAF-15.EGFP in cell lysates, the lower panel shows the expression of Flag-tagged proteins. kD, kilodalton. Full-length blots are presented in Supplementary Fig. 4. (**b**) VHL-1 impairs mTORC1 activity in *C.* *elegans*. Western blot analysis of phosphorylation of p70 S6 kinase in wild type and *vhl-1(ok161)* mutant *C. elegans* animals (upper panel). The graph shows quantification of phospho p70 S6K levels normalized to β-actin from three independent experiments. Mean ± SEM, *p* < 0.05 (*t* test). kD, kilodalton. Full-length blots are presented in Supplementary Fig. 4. (**c**) *daf-15/Raptor* expression was not altered in *vhl-1* and *hif-1* mutants. *daf-15/Raptor* mRNA expression in wild type animals, *vhl-1(ok161),* and *hif-1(ia4)* mutants was analyzed by qPCR. Data are mean ± SEM of four independent experiments. *t* test. n.s., not significant. (**d**-**h**) Loss of *vhl-1* suspends larval development defects of *daf-15/Raptor* deficient *C.* *elegans* animals. *rrf-3* control animals and *rrf-3;vhl-1* mutants were fed with *daf-15/Raptor* RNAi and control RNAi respectively. (**d**) Quantification of arrested L3 larvae. The total number of L3 larvae and the portion that progressed development to adulthood was counted. n > 300 for each condition. Graph shows mean ± SEM. *t* test, ***p < 0.001 versus *rrf-3* on control RNAi. (**e**–**h**) Representative images, scale bar indicates 1 mm. (**i**-**m**) Mutation of *vhl-1* prevents autophagy. In wild type (WT) (**j**) and *vhl-1* mutant (**k**) animals mCherry::LGG-1 is diffusely distributed in the cytosol. After starvation, the number of intestinal mCherry::LGG-1 punctae is strongly increased in wild type (**l**). Loss of *vhl-1* reduces intestinal LGG-1 foci (**m**). Representative fluorescence images of young adult animals expressing mCherry::LGG-1 in the intestine. Scale bar indicates 10 µm. (**i**) Quantification of mCherry::LGG-1 foci in intestinal cells. Box plot marks median and first and third quartiles, whiskers extend to 10th and 90th percentile, and outliers are plotted as points. n > 50 for each condition. ***p < 0.001; n.s., not significant (ANOVA).

First, we analyzed whether the direct association between VHL and RAPTOR is conserved between species. Co-immunoprecipitation experiments from HEK293T cells transiently transfected with DAF-15, the *C.* *elegans* homolog of human RAPTOR, and VHL-1 were performed. Indeed, DAF-15/RAPTOR co-precipitated with VHL-1 (Fig. 4a). To investigate the functional connection between VHL and mTORC1 signaling we analyzed canonical mTORC1 downstream targets. Monitoring the phosphorylation status of p70 S6 kinase has been previously utilized to characterize mTORC1 activity in *C.* *elegans*^33,34^*.* Inactivation of *vhl-1* enhanced the phosphorylation level of p70 S6K compared to wild type (Fig. 4b) consistent with hyperactivation of mTORC1 in *vhl-1* mutants. We next examined whether VHL-1 might regulate *daf-15/Raptor* transcription by performing quantitative PCR. The mRNA expression of *daf-15/Raptor* was not changed in *vhl-1* and *hif-1* mutants compared to wild type (Fig. 4c). Hence, the activation of the mTORC1 pathway in *vhl-1* mutants was not due alteration of corresponding *daf-15/Raptor* mRNA levels.

mTORC1 is essential for larval growth and development in *C.* *elegans* and previous studies have shown that loss of *daf-15/Raptor* causes developmental arrest at L3 larval-stage^35^. We inactivated *daf-15/Raptor* and *vhl-1* together and found that a substantial portion of larvae (approx. 42%) progressed through development to adulthood, while all larvae from *daf-15/Raptor* deficient animals arrested at L3 stage (Fig. 4d, f, h). *vhl-1* single mutants did not display growth defects (Fig. 4g). The observation that mutation of *vhl-1* suppresses the developmental defects associated with loss of *daf-15/Raptor* suggests that the genes function in the same pathway. mTORC1 is a well-described inhibitor of autophagy, a cellular recycling pathway degrading cytoplasmic material. LGG-1, the *C.* *elegans* homolog of mammalian LC3 has been widely used as an indicator of autophagy^36,37^. To monitor the function of *vhl-1* in autophagy we used a reporter strain expressing mCherry-tagged LGG-1 in the intestine^38^. Upon induction of autophagy, mCherry::LGG-1 changes its diffuse cytoplasmic distribution pattern to punctate structures reflecting autophagosomal localization of LGG-1. As expected, mCherry::LGG-1 was diffusely localized in intestinal cells of fully fed wild type worms and relocalized into punctae after starvation, a potent inducer of intestinal autophagosome formation (Fig. 4i,j,l). Well-fed *vhl-1* mutants also displayed a diffuse cytoplasmic LGG-1 distribution (Fig. 4k). Importantly, upon starvation we observed a significant decrease of mCherry::LGG-1 positive foci in *vhl-1* mutants compared to wild type (Fig. 4i,l,m). Together, these results provide strong evidence for the evolutionary conservation of the link between VHL and mTORC1 signaling and indicate that VHL-1 serves as an inhibitor for mTORC1 in multiple species.

### RAPTOR is a novel target of the VHL E3 ubiquitin ligase

**Figure 5:**
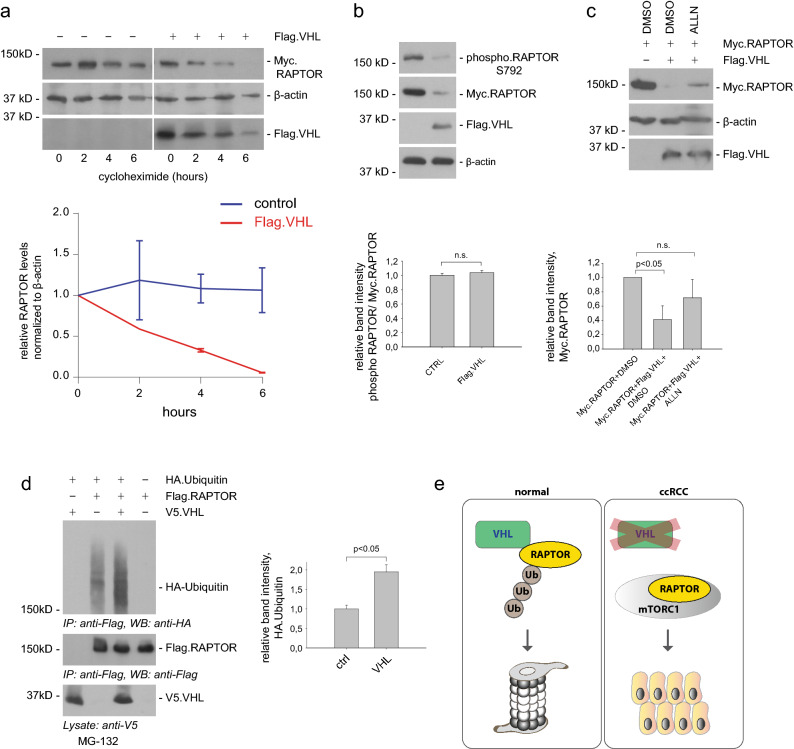
VHL leads to ubiquitination of RAPTOR. (**a**) VHL impairs RAPTOR protein stability. HEK293T cells were transfected with plasmids expressing Flag-VHL and Myc-RAPTOR. 24 h after transfection cells were treated with cycloheximide (40 μg/mL) for the indicated times. The levels of VHL and RAPTOR were monitored by Western blot analysis. β-actin levels were used as a loading control. Lower panel shows relative RAPTOR levels normalized to β-actin. Full-length blots are presented in Supplementary Fig. 4. (**b**) RAPTOR phosphorylation at Serine 792 is independent of VHL. 293 T cells were transiently transfected with Flag.VHL and Myc. RAPTOR. Lysates were analyzed by immunoblotting for anti- phospho RAPTOR S792, anti-Myc and anti-Flag antibody. β-actin was used as loading control. Quantification of three independent experiments. *t* test; mean values ± SEM. n.s., not significant. Full-length blots are presented in Supplementary Fig. 4. (**c**) Proteasome inhibition prevents RAPTOR degradation by VHL. HEK293T cells were co-transfected with Myc.RAPTOR and Flag.VHL and treated with the proteasome inhibitor ALLN (30 µM, overnight) as indicated. DMSO was used as vehicle control. Cell lysates were subjected to immunoblotting using anti-Flag and anti-Myc antibody. Quantification of three independent experiments. *t* test; mean values ± SEM. n.s., not significant. Full-length blots are presented in Supplementary Fig. 4. (**d**) Polyubiquitinylation of RAPTOR is induced by VHL. HEK293T cells were transfected with HA.Ubiquitin, Flag.RAPTOR, and V5.VHL as indicated. After transfection the cells were treated with the proteasome inhibitor MG132 (5 µM) for 12 h. Flag.RAPTOR was immunoprecipitated with anti-Flag antibody (middle panel) and analyzed for co-precipitated Ubiquitin with anti-HA antibody (upper panel). Cell lysates were used for detecting expression of VHL by anti-V5 antibody (lower panel). Quantification of three independent experiments. *t* test; mean values ± SEM. Full-length blots are presented in Supplementary Fig. 4. (**e**) Model depicts the modulation of RAPTOR/mTORC1 by VHL. VHL interacts with RAPTOR and promotes RAPTOR degradation by ubiquitination. Loss of VHL enhances RAPTOR protein stability, thereby facilitating mTORC1 signaling and tumor proliferation.

Our results indicate that VHL regulates RAPTOR protein abundance. Next, we sought to investigate the underlying mechanism. First, the decay kinetic of RAPTOR protein after treatment with the translation inhibitor cycloheximide was analyzed. Upon VHL-overexpression RAPTOR levels were significantly diminished with time by cycloheximide treatment, while RAPTOR abundance was unchanged under basal conditions (Fig. 5a). Previous work has shown that AMPK phosphorylates RAPTOR, induces binding to 14–3–3 and inhibits mTORC1 activity^39^. VHL also associated with AMPK (Supplementary Fig. 1b) but did not interfere with AMPK-mediated RAPTOR phosphorylation (Fig. 5b). VHL functions as the substrate recognition subunit of an E3 ubiquitin ligase complex targeting HIFα and other proteins for proteasomal degradation. To test whether the regulation of RAPTOR by VHL depends on the function of the proteasome, RAPTOR levels following treatment with the proteasome inhibitor ALLN were analyzed. While overexpression of VHL strongly suppressed RAPTOR protein levels, inhibition of the proteasome by ALLN prevented VHL-mediated RAPTOR degradation (Fig. 5c). These data suggest that RAPTOR could be a specific target of the VHL ubiquitin ligase complex. Therefore, we next examined whether RAPTOR is ubiquitinated by VHL. Flag-tagged RAPTOR, V5-tagged VHL, and HA-ubiquitin were transfected into HEK293 cells. We detected RAPTOR polyubiquitination, and VHL overexpression further increased ubiquitination of RAPTOR (Fig. 5d). Together our data reveal a novel mechanism where VHL increases ubiquitination and degradation of RAPTOR and thereby limits mTORC1 signaling (Fig. 5e).

## Discussion

Hyperactivation of mTORC1 signaling in *VHL*-deficient ccRCC is well described in the literature^6,7^ although the functional inter-connection between VHL and mTORC1 and their mutual regulation are still ill defined. Integrating biochemical assays with molecular renal cancer cell analyses we show here that VHL binds to the key subunits of mTORC1, limits RAPTOR protein abundance, and suppresses mTORC1 signaling. Importantly, our *C.* *elegans* analyses demonstrate that the link between VHL and RAPTOR/mTORC1 is conserved from nematodes to human. We propose a new mechanism by which VHL regulates mTORC1 signaling: VHL-mediated ubiquitinylation and degradation of RAPTOR may result in impaired mTORC1 activity.

Our investigation identified RAPTOR as a new VHL substrate in ccRCC. The endogenous levels of RAPTOR were elevated in *VHL*-deficient ccRCC cells and *VHL* shRNA cells (Figs. 2d, 3a). The in vivo significance of our findings was corroborated by increased RAPTOR expression in ccRCC tumor samples (Fig. 1d). Conversely, transient over-expression of VHL resulted in a concomitant decrease in RAPTOR protein (Fig. 2a-c). The near complete depletion of RAPTOR following treatment with cycloheximide within 6 h strongly suggests that VHL regulation occurs at the post-translational level (Fig. 5a), while mRNA expression of *RPTOR* was not affected in different cell lines (Fig. 3b,c). Moreover the regulation of RAPTOR by VHL was HIFα independent as pharmacological inhibition of HIFα did not alter RAPTOR abundance in ccRCC cells (Fig. 3g,h and Supplementary Fig. 3d,e,f). Inhibiting the proteasome partially rescued RAPTOR levels (Fig. 5c). That RAPTOR is ubiquitylated by the VHL-associated E3 ligase is demonstrated by our finding that overexpression of VHL increases its ubiquitylation (Fig. 5d). Our data demonstrate VHL’s ability to recognize and ubiquitinylate RAPTOR and consequently to target it for proteasomal degradation. Of note, RAPTOR level has been shown previously to correlate with mTORC1 activity^40,41^. VHL has been reported to regulate additional oncogenic substrates besides the well-established HIFs such as ZHX2^42^, AKT^12^ and SFMBT1^43^. Hence the function of VHL appears to be multi-faced, including both E3 ligase-dependent and -independent functions.

Post-translational modification of mTOR pathway members by ubiquitinylation has been shown to modulate mTOR signaling activity. mTOR, the essential kinase of both mTORC1 and mTORC2 complexes, is targeted for ubiquitinylation and degradation by binding to the tumor suppressor FBXW7^44^. In colorectal carcinoma, downregulation of the E3 ligase FBX8 correlated with enhanced mTOR activity and might thereby promote invasion and metastasis^45^. RICTOR, the integral component of mTORC2, is also degraded through an FBXW7-mediated ubiquitination/proteasome mechanism^46^. RAPTOR protein is reportedly modified by ubiquitylation^47–49^, but the mechanism how the ubiquitin pathway governs RAPTOR/mTORC1 activation remains unclear. The DDB1-CUL4 ubiquitin ligase complex has been shown to interact with RAPTOR, but it seems to impact upon mTORC1 signaling indirectly through a non-degradative mechanism by affecting the assembly of the mTORC1 complex^50^. In this study we uncovered a novel VHL-mediated regulation of mTORC1 by targeted RAPTOR ubiquitination and degradation.

Our data support the notion that VHL30 and VHL19 have different functional specializations. We connect full length VHL30 with RAPTOR and mTORC1 pathway regulation while VHL19 lacks this interaction (Fig. 2c). Both isoforms act as tumor suppressor inhibiting cancer development when a wild type copy is reintroduced in ccRCC, but isoform-specific VHL functions are emerging from the literature^51^. VHL30 has been shown to interact with p53 and p14ARF^51,52^ to control cell cycle and apoptosis. Moreover, VHL30 co-localizes predominantly with cytoplasmic microtubules and alters microtubule dynamics^53^. Our findings show that VHL30 executes RAPTOR regulating functions, suggesting that VHL30 may contribute independently to tumor suppression in specific contexts. Moreover, we found that VHL mutations with altered binding capacity to HIFα^25,54^ differentially regulated RAPTOR protein levels. Particularly, the VHL mutant Y112N did not lead to RAPTOR destabilization, in contrast to the VHL mutants Y98N, Y98H and Y112H which reduced RAPTOR protein comparable to wild type VHL (Fig. 2e). The mutational hot spot residues Y98 and Y112 make important contributions to HIFα binding^55,56^ and have been shown to affect substrate interactions to different extents with type 2B mutations (Y98N and Y112N) causing more severe defects and reduced ubiquitylation activity^25^. The binding of other ubiquitinylation targets to the same key surface of VHL may be affected likewise. In our study VHL mutants still associated with RAPTOR (Supplementary Fig. 2) albeit overexpression of VHL mutants could mask differences in binding affinity and activity. This remains to be determined in a more rigorous approach. Nevertheless, the effect of VHL on RAPTOR/mTORC1 complex stability suggests an additional mechanism for tumorigenesis in VHL-dependent ccRCC and part of the phenotypic variability observed in VHL disease may be due differential impact of *VHL* mutations on oncogenic pathways.

Remarkably, the mechanism of RAPTOR/mTORC1 inhibition by VHL is conserved in evolution between nematodes and mammals. Consistent with the hyperactivation of the mTORC1 pathway in *VHL-*deficient ccRCC cells, *vhl-1 C.* *elegans* mutants displayed increased phosphorylation of ribosomal S6 kinase, which represents the main activity of the mTORC1 pathway (Fig. 4b). The primary role of mTORC1 is to promote growth-related processes and development as well as to inhibit autophagy. In fact developmental defects of *Raptor/daf-15* deficient worms were recovered by knockdown of *vhl-1* (Fig. 4d-h) and starvation-induced autophagy was markedly suppressed by inactivation of *vhl-1* (Fig. 4i,l,m). Together, our *C.* *elegans* findings support the inhibitory function of VHL in mTORC1 pathway regulation. The nematode *C.* *elegans* has been widely used to elucidate the molecular function of mTOR signaling proteins and their hierarchical order in the pathways^29^. *C.* *elegans* overcomes many problems of VHL-studies in cell culture and mouse models and offers a powerful tool in studying molecular and genetic aspects of the VHL-mTOR network^32^. Exploiting the genetic screening possible in *C. elegans* might provide further insight how mTOR influences phenotype and disease progression with VHL, and potentially reveal novel concepts to treat VHL-driven cancer.

Studies during the last decade have highlighted that mTORC1 signaling is hyperactivated in ccRCC. mTORC1 hyperactivation correlates with clinico-pathological parameters and poor outcome in ccRCC patients. The induction of mTORC1 signaling in ccRCC may occur at several levels and by multiple mechanisms: First, genomic studies have identified genetic alterations activating the mTORC1 pathway including *MTOR, PTEN, AKT*, and *PIK3CA* in ~ 26% of ccRCC cases^2^. Second, HIF-mediated aberrant expression of mTORC1 upstream genes including the mTOR inhibitor *DEPTOR*^16^, *REDD1*^13,14^ and the amino acid carrier *SLC7A5*^15^ has been shown to modulate mTORC1 signaling activity. Next, essential HIF-independent mechanisms have also been described recently. VHL can directly bind and inhibit AKT kinase activity. In *VHL*-deficient cells AKT was activated, promoting cell survival and tumorigenesis^12^. In this study we describe a novel VHL mechanism for regulation of mTORC1 signaling in vitro and in vivo by limiting RAPTOR protein abundance. The relative contribution of these pathways to mTORC1 activation in ccRCC remains to be determined.

## Material and methods

### Reagents and plasmids

Cycloheximide was obtained from Sigma, ALLN from Calbiochem, MG-132 from Enzo Life Sciences, DMOG from Frontier Scientific. PT2385 was purchased from Abcam and IDF-11774 from Hycultec. All reagents were used at concentrations as indicated.

Myc.RAPTOR was a gift from David Sabatinini (Addgene plasmid # 1859)^40^ and RAPTOR cDNA was fused by standard cloning techniques to a pcDNA6 vector encoding an N-terminal GFP or Flag-tag (Invitrogen). pcDNA3-Flag mTOR wt was a gift from Jie Chen (Addgene plasmid # 26603)^57^. VHL30 was cloned into pcDNA6 vector with N-terminal Flag-, GFP- and V5-tag, and C-terminal RFP-tag. VHL(AA1-53) and VHL19 were generated by standard cloning techniques and fused to the appropriate vectors. Full-length human VHL(Y98N), VHL(Y112N), VHL(Y98H) und VHL(Y112H) were generated by site-directed mutagenesis. The resulting constructs were sequence verified. For Flag-tagged and V5-tagged Luciferase the Luciferase cDNA (Addgene Plasmid #13458) was cloned into Flag- and V5.pcDNA6 vector, respectively (Invitrogen). The Flag-tagged constructs for GFP and MO-25 were previously described^58^, as well as the constructs for HA.Ubiquitin and CD2AP^59^. LST8, PRAS40, AMPK and DEPTOR were subcloned by PCR from human cDNA (Agilent), and fused to the V5-tagged pcDNA6 vector (Invitrogen). DAF-15.pEGFP was kindly provided by Ralf Baumeister, *C. elegans* VHL-1 with Flag-tag in pcDNA6 was cloned using cDNA from N2 animals.

### Antibodies

Antibodies used in this study included antibody to Flag (Sigma), antibody to Myc (Clone 9E10 Santa Cruz Biotechnology), mouse antibody to GFP (B-2, Santa Cruz Biotechnology), rabbit antibody to GFP (MBL), antibody to RAPTOR (Cell Signaling), rabbit antibody to VHL (Santa Cruz Biotechnology FL-181 and Cell Signaling), antibody to β-actin (Sigma), antibody to γ-tubulin (Sigma), rabbit antibody to V5 (Millipore), and mouse antibody to HA Clone12CA5 (Roche). The antibodies to mTOR, p70 S6 kinase, phospho-p70 S6 Kinase, phospho-S6 ribosomal protein, phospho-RAPTOR S792, REDD1 and phospho-drosophila p70 S6 (Thr398) kinase were all obtained from Cell Signaling, the antibody against HIF1α from BD Transduction Laboratories and the antibody against HIF2α from Abcam.

### Cell culture and transfections

HEK 293 T and HeLa cells were grown in Dulbecco’s modified Eagle’s medium (DMEM) supplemented with 10% FBS. For 293 T cells, transient transfections were carried out using the calcium phosphate method; and cells were lysed 24 h after transfection in IP-buffer containing 20 mM Tris·HCl (pH 7,5), 1% Triton X-100, 50 mM NaF, 15 mM Na4P2O7, 0.1 mM EDTA, 50 mM NaCl, 2 mM Na3VO4, and cOmplete protease inhibitor (Roche) as described in reference^60^. HeLa cells were transfected with Lipofectamine 3000 (Invitrogen).

A498 and RPTEC cells were obtained from ATCC, RPTEC cells were cultured in DMEMF12 supplemented with hTERT Immortalized RPTEC Growth Kit (ATCC) and G418*. VHL*-negative 786-O and RCC4 cells, as well as 786-O and RCC4 cells expressing VHL were previously described^61,62^ and were cultured in DMEM (RCC4) or RPMI­1640 (786-O) medium supplemented with 10%FBS and Geniticin 0.5 mg/ml.

For immunoprecipitation experiments, cells were transfected with the indicated plasmids, washed with PBS, lysed in IP buffer and after centrifugation (13.000 rpm, 15 min, 4 °C followed by 43,000 rpm, 30 min, 4 °C) the supernatants were incubated with anti-FLAG M2 affinity beads (Sigma) or V5 beads (Abcam) overnight at 4 °C. For GFP-tagged protein, lysates were incubated with GPF antibody (MBL) overnight, followed by incubation with Protein A beads (GE Healthcare) for 2 h at 4 °C. All the precipitates were washed extensively with IP buffer and subjected to SDS-PAGE and immunoblotting analysis with anti-Flag, anti-myc, anti-V5 or anti-GFP antibodies.

Immunoprecipitation experiments from endogenous proteins were previously described^63^. Briefly, for detection of the interaction between VHL and RAPTOR, after lysis with IP-buffer four dishes of confluent HeLa cells were pooled for each condition. After centrifugation (13.000 rpm, 15 min, 4 °C followed by 43,000 rpm, 30 min, 4 °C) the supernatants were incubated with anti-VHL antibody (Santa Cruz Biotechnology) or normal rabbit IgG (Santa Cruz Biotechnology) overnight. After addition and incubation with Protein A beads (GE Healthcare) for 2 h, 4 °C, the precipitates were washed and analyzed by Western blot with antibodies against RAPTOR and VHL.

For the quantification of total amounts of the overexpressed and endogenous proteins, cells were split in parallel, lysed in the buffer described above and analyzed by immunoblotting. The blots were scanned and bands were quantified using LabImage 1D software. Data were expressed as means or as means ± SEM. The statistical analysis was performed using SigmaPlot 11.0 software.

To analyze the turnover of RAPTOR, 24 h after transfection cells were treated with cycloheximide in DMEM for the indicated time points followed by lysis in IP-buffer as described in reference^60^. Proteins were fractionated by SDS/PAGE, and protein levels were analyzed by Western blot.

Ubiquitination assays were performed as previously described^59^. Briefly, HEK 293 T cells were transfected with the plasmids as indicated along with the HA-tagged ubiquitin construct and treated with MG-132 (5 µM, overnight). 24 h after transfection, the cells were washed with PBS and lysed in RIPA buffer (1% Triton X-100, 0.5% sodium deoxycholate, 0.1% SDS, 150 mM NaCl, 50 mM NaF, 2 mM EDTA, 13.7 mM Na2HPO4, 6.3 mM NaH2PO4). The lysates were clarified by ultracentrifugation and incubated with Flag-M2 affinity beads at 4 °C for 2 h. After washing extensively with RIPA buffer, precipitates were subjected to SDS-PAGE and immunoblotting analysis with anti-Flag, anti-V5 and anti-HA antibodies.

### shRNA stable polyclonal cell line

To generate a HeLa cell line for tetracycline-inducible knockdown of VHL, a lentivirus-based transduction system (pLVTH) was used as previously described^64^. The VHL shRNA targeting sequence was 5’ ACACAGGAGCGCATTGCACAT3’, the control targeting sequence was 5′ GTACGCGGAATACTTCGA3’.

### Quantitative real-time PCR

Total RNA was obtained from HeLa cells using RNeasy Mini Kit (Qiagen) and reverse-transcribed using the SuperScript™ IV First-Strand Synthesis System (Invitrogen) according to the manufacturer’s protocol. qRT–PCR was performed on a LightCycler 480 (LC 480, Roche). GAPDH or HSPCB were used as normalization controls. Each biological replicate was measured in technical triplicates. The primers used for qRT–PCR were: *VHL*: 5′CACAGCTACCGAGGTCAC3′ and 3′CTGAATTATTTGTGCCATCTCTCA5′, *RAPTOR*_primer 1: 5′GATCGCATGTGGCTCCGT3′ and 3′TCAACAACATCAAGTACTACGACG5′, *RAPTOR*:_primer 2: 5′ACACCAGAATCTTCCAGAA3′ and 3′AGTCCTTCAACTCAATTCTTAC5′, *PAI-1:* 5′CCTGGTTCTGCCCAAGTTCT3′ and 3′ ATCGAGGTGAACGAGAGTGG5′, *HSPCB:* 5′TCTGGGTATCGGAAAGCAAGCC3V and 3′CAAGATGCCTGAGGAAGTGCAC5′, *GAPDH:* 5′CATTTCCTGGTATGACAA3′ and 3′CAAGAGGAAGAGAGAGAC5′.

For *C. elegans* samples, total RNA was isolated from L4 worms using TRI Reagent (Sigma-Aldrich) and a RNA clean and concentrator kit (Zymo Research Corp.). DNase treatment was performed using the on-column DNase digestion (Qiagen). To generate cDNA 1 μg of RNA was reverse transcribed with oligo-dT primer and SuperScript™ IV First-Strand Synthesis System (Invitrogen). qPCR reactions were performed in at least three independent samples in triplicates. Results were normalized against endogenous *cdc-42* and *Y45F10D.4* expression. Primer sequences for *daf-15* are: 5′TGAGTGGAAGAATGTCAT3′ and 3′CATCAAATGAGACTGCTC5′.

The quantification of changes in mRNA expression levels was based on 2^−ΔΔCt^ method.

### Immunofluorescence and imaging

HeLa cells were seeded onto poly-l-lysine (Sigma) coated coverslips. 24 h after transfection the cells were fixed in 4% paraformaldehyde/PBS. After permeabilization and blocking in PBS containing 0,1% Triton X-100 and 1% fish gelatine cells were stained for 10 min with Hoechst 33,342 (Molecular Probes). Coverslips were mounted onto slides using ProLong Diamond Antifade Mountant (Invitrogen), and visualized under a Zeiss Axiovert 200 M2-microscope as previously described^60^.

### *C. elegans* growth conditions

Unless otherwise indicated *C.* *elegans* were cultured at 20 °C on standard NGM plates seeded with E. coli OP50. The strains used were as follows: wild type N2, CB5602 *vhl-1(ok161),* NL2099 *rrf-3(pk1426),* ENH626 *rrf-3(pk1426);vhl-1(ok161),* ZG31 *hif-1(ia4),* BR7019 *byIs205[Pnhx-2::mCherry::lgg-1],* ENH672 *vhl-1(ok161);byIs205[Pnhx-2::mCherry::lgg-1].*

### RNAi

RNAi experiments were carried out as described^65^. HT115 bacteria transformed with *daf-15/Raptor* RNAi or empty vector (pL4440) were grown overnight in 12.5 μg/ml tetracycline and 50 μg/ml ampicillin. The following day, cultures were diluted 1:10 and grown to an OD600 of 0.8–1.0 and induced with 0.7 mM IPTG. This culture was seeded on NGM plates containing tetracycline, ampicillin, and 1 mM IPTG. The RNAi plasmid for knockdown of *daf-15/Raptor* was derived from the Ahringer RNAi library and confirmed by sequencing.

### Larval arrest test

*rrf-3* and *rrf-3;vhl-1* mutants were fed with *daf-15/Raptor* or control L4440 RNAi. To obtain synchronized progeny, adult animals were allowed to lay eggs for 2-4 h on RNAi plates. The total number of eggs was counted after removing parents. Arrested L3 larvae and adult animals were counted after 72 h. All experiments were performed at 20 °C.

### Autophagy assay

A genomically integrated version of an intestinal mCherry::LGG-1 marker was used^38,66^. Animals were raised at 20 °C until young adult. Worms were then either placed on empty NGM plates or kept on OP50 seeded plates for 3 h. To assess autophagy, the posterior part of young adult animals was imaged with an Axioplan 2 microscope at high magnification (630x). mCherry-positive punctae within one int9 cell per animal were counted using ImageJ as described^66^. Three independent biological samples were analyzed for autophagic events. ANOVA test was performed with SigmaStat 3.5.

### Analysis of p 70 S6K phosphorylation in* C. elegans*

To prepare *C.* *elegans* proteins, L4 stage wild type and *vhl-1(ok161)* animals were collected and mechanically disrupted using an electronic hand homogenizer (IKA T10, ULTRA-TURRAX) in lysis buffer (50mMTris-HCl [pH 7.5], 150 mM NaCl, 1 mM EDTA, 0.5% NP-40, phosphatase inhibitors, and protease inhibitors). Phosphorylation of p70 S6K was detected by Western blot and levels were analyzed using the drosophila phospho p70 S6 Kinase antibody (Cell Signaling)^33^. Anti-actin (Sigma) was used to verify equivalent input of total protein.

### Cell viability assay

6 h after transient transfection, 293 T cells were counted and seeded in at least triplicates in 96-well-plates (approx. 5 × 10^4^ cells/well). RCC4 cells were counted and seeded 24 h after last splitting (approx. 1 × 100^4^ cells/well). Viability was assessed using the MTT Cell Proliferation Assay Kit (Cayman Chemical) performed according to the manufacturer’s protocol. Viability was calculated relative to control.

## Supplementary Information

Supplementary Information.

## References

[1] Global Burden of Disease Cancer, C., *et al.* Global, regional, and national cancer incidence, mortality, years of life lost, years lived with disability, and disability-adjusted life-years for 29 cancer groups, 1990 to 2017: A systematic analysis for the global burden of disease study. *JAMA Oncol.* **5**, 1749–1768 (2019). 10.1001/jamaoncol.2019.2996 PMC6777271 31560378

[2] Clark, D.J.*, et al.* Integrated proteogenomic characterization of clear cell renal cell carcinoma. *Cell* **179**, 964–983 e931 (2019). 10.1016/j.cell.2019.10.007 PMC7331093 31675502

[3] Ricketts CJ (2018). The Cancer genome atlas comprehensive molecular characterization of renal cell carcinoma. Cell Rep..

[4] Cancer Genome Atlas Research, N. Comprehensive molecular characterization of clear cell renal cell carcinoma. *Nature* **499**, 43–49 (2013). 10.1038/nature12222 PMC3771322 23792563

[5] Sato Y (2013). Integrated molecular analysis of clear-cell renal cell carcinoma. Nat. Genet..

[6] Pantuck AJ (2007). Prognostic relevance of the mTOR pathway in renal cell carcinoma: Implications for molecular patient selection for targeted therapy. Cancer.

[7] Robb VA, Karbowniczek M, Klein-Szanto AJ, Henske EP (2007). Activation of the mTOR signaling pathway in renal clear cell carcinoma. J. Urol..

[8] Ilagan E, Manning BD (2016). Emerging role of mTOR in the response to cancer therapeutics. Trends in cancer.

[9] Cornu M, Albert V, Hall MN (2013). mTOR in aging, metabolism, and cancer. Curr. Opin. Genet. Dev..

[10] Liu GY, Sabatini DM (2020). mTOR at the nexus of nutrition, growth, ageing and disease. Nat. Rev. Mol. Cell Biol..

[11] Zhang, Y.*, et al.* A Pan-Cancer Proteogenomic Atlas of PI3K/AKT/mTOR Pathway Alterations. *Cancer cell* **31**, 820–832 e823 (2017). 10.1016/j.ccell.2017.04.013 PMC5502825 28528867

[12] Guo J (2016). pVHL suppresses kinase activity of Akt in a proline-hydroxylation-dependent manner. Science.

[13] Brugarolas J (2004). Regulation of mTOR function in response to hypoxia by REDD1 and the TSC1/TSC2 tumor suppressor complex. Genes Dev..

[14] Kucejova B (2011). Interplay between pVHL and mTORC1 pathways in clear-cell renal cell carcinoma. Mol. Cancer Res. MCR.

[15] Elorza A (2012). HIF2alpha acts as an mTORC1 activator through the amino acid carrier SLC7A5. Mol. Cell.

[16] Doan H (2019). HIF-mediated suppression of DEPTOR confers resistance to mTOR kinase inhibition in renal cancer. iScience.

[17] Chen F, Chandrashekar DS, Varambally S, Creighton CJ (2019). Pan-cancer molecular subtypes revealed by mass-spectrometry-based proteomic characterization of more than 500 human cancers. Nat. Commun..

[18] Iliopoulos O, Kibel A, Gray S, Kaelin WG (1995). Tumour suppression by the human von Hippel–Lindau gene product. Nat. Med..

[19] Kibel A, Iliopoulos O, DeCaprio JA, Kaelin WG (1995). Binding of the von Hippel–Lindau tumor suppressor protein to Elongin B and C. Science.

[20] Iwai K (1999). Identification of the von Hippel–Lindau tumor-suppressor protein as part of an active E3 ubiquitin ligase complex. Proc. Natl. Acad. Sci. U.S.A..

[21] Sancak Y (2008). The Rag GTPases bind raptor and mediate amino acid signaling to mTORC1. Science.

[22] Yadav RB (2013). mTOR direct interactions with Rheb-GTPase and raptor: Sub-cellular localization using fluorescence lifetime imaging. BMC Cell Biol..

[23] Schoenfeld AR, Davidowitz EJ, Burk RD (2001). Endoplasmic reticulum/cytosolic localization of von Hippel–Lindau gene products is mediated by a 64-amino acid region. Int. J. Cancer.

[24] Gossage L, Eisen T, Maher ER (2015). VHL, the story of a tumour suppressor gene. Nat. Rev. Cancer.

[25] Knauth K, Bex C, Jemth P, Buchberger A (2006). Renal cell carcinoma risk in type 2 von Hippel–Lindau disease correlates with defects in pVHL stability and HIF-1alpha interactions. Oncogene.

[26] Choueiri TK, Kaelin WG (2020). Targeting the HIF2-VEGF axis in renal cell carcinoma. Nat. Med..

[27] Wallace EM (2016). A small-molecule antagonist of HIF2alpha Is efficacious in preclinical models of renal cell carcinoma. Can. Res..

[28] Ban HS (2017). The novel hypoxia-inducible factor-1alpha inhibitor IDF-11774 regulates cancer metabolism, thereby suppressing tumor growth. Cell Death Dis..

[29] Blackwell TK, Sewell AK, Wu Z, Han M (2019). TOR signaling in *Caenorhabditis elegans* development, metabolism, and aging. Genetics.

[30] Muller RU (2009). The von Hippel Lindau tumor suppressor limits longevity. J. Am. Soc. Nephrol..

[31] Mehta R (2009). Proteasomal regulation of the hypoxic response modulates aging in *C. elegans*. Science.

[32] Ganner A, Neumann-Haefelin E (2017). Genetic kidney diseases: *Caenorhabditis elegans* as model system. Cell Tissue Res..

[33] Heintz C (2017). Splicing factor 1 modulates dietary restriction and TORC1 pathway longevity in *C. elegans*. Nature.

[34] Nakamura S (2016). Mondo complexes regulate TFEB via TOR inhibition to promote longevity in response to gonadal signals. Nat. Commun..

[35] Jia K, Chen D, Riddle DL (2004). The TOR pathway interacts with the insulin signaling pathway to regulate *C. elegans* larval development, metabolism and life span. Development.

[36] Hansen M (2008). A role for autophagy in the extension of lifespan by dietary restriction in *C. elegans*. PLoS Genet..

[37] Feng Y, He D, Yao Z, Klionsky DJ (2014). The machinery of macroautophagy. Cell Res..

[38] Gosai SJ (2010). Automated high-content live animal drug screening using *C. elegans* expressing the aggregation prone serpin alpha1-antitrypsin Z. PloS One.

[39] Gwinn DM (2008). AMPK phosphorylation of raptor mediates a metabolic checkpoint. Mol. Cell.

[40] Sarbassov DD (2004). Rictor, a novel binding partner of mTOR, defines a rapamycin-insensitive and raptor-independent pathway that regulates the cytoskeleton. Curr. Biol.CB.

[41] Dalle Pezze P (2012). A dynamic network model of mTOR signaling reveals TSC-independent mTORC2 regulation. Sci. Signal..

[42] Zhang J (2018). VHL substrate transcription factor ZHX2 as an oncogenic driver in clear cell renal cell carcinoma. Science.

[43] Liu, X.*, et al.* Genome-wide screening identifies SFMBT1 as an oncogenic driver in cancer with VHL loss. *Molecular cell* **77**, 1294–1306 e1295 (2020). 10.1016/j.molcel.2020.01.009 PMC7093231 32023483

[44] Mao JH (2008). FBXW7 targets mTOR for degradation and cooperates with PTEN in tumor suppression. Science.

[45] Wang FF (2017). FBX8 is a metastasis suppressor downstream of miR-223 and targeting mTOR for degradation in colorectal carcinoma. Cancer Lett..

[46] Koo J, Wu X, Mao Z, Khuri FR, Sun SY (2015). Rictor undergoes glycogen synthase kinase 3 (GSK3)-dependent, FBXW7-mediated ubiquitination and proteasomal degradation. J. Biol. Chem..

[47] Choi SI, Maeng YS, Kim KS, Kim TI, Kim EK (2014). Autophagy is induced by raptor degradation via the ubiquitin/proteasome system in granular corneal dystrophy type 2. Biochem. Biophys. Res. Commun..

[48] Hussain S (2013). Ubiquitin hydrolase UCH-L1 destabilizes mTOR complex 1 by antagonizing DDB1-CUL4-mediated ubiquitination of raptor. Mol. Cell. Biol..

[49] Wang B (2017). TRAF2 and OTUD7B govern a ubiquitin-dependent switch that regulates mTORC2 signalling. Nature.

[50] Ghosh P, Wu M, Zhang H, Sun H (2008). mTORC1 signaling requires proteasomal function and the involvement of CUL4-DDB1 ubiquitin E3 ligase. Cell Cycle.

[51] Minervini G (2015). Isoform-specific interactions of the von Hippel–Lindau tumor suppressor protein. Sci. Rep..

[52] Roe JS (2006). p53 stabilization and transactivation by a von Hippel–Lindau protein. Mol. Cell.

[53] Frew IJ, Smole Z, Thoma CR, Krek W (2013). Genetic deletion of the long isoform of the von Hippel–Lindau tumour suppressor gene product alters microtubule dynamics. Eur. J. Cancer.

[54] Cockman ME (2000). Hypoxia inducible factor-alpha binding and ubiquitylation by the von Hippel–Lindau tumor suppressor protein. J. Biol. Chem..

[55] Hon WC (2002). Structural basis for the recognition of hydroxyproline in HIF-1 alpha by pVHL. Nature.

[56] Min JH (2002). Structure of an HIF-1alpha -pVHL complex: Hydroxyproline recognition in signaling. Science.

[57] Vilella-Bach M, Nuzzi P, Fang Y, Chen J (1999). The FKBP12-rapamycin-binding domain is required for FKBP12-rapamycin-associated protein kinase activity and G1 progression. J. Biol. Chem..

[58] Viau, A.*, et al.* Cilia-localized LKB1 regulates chemokine signaling, macrophage recruitment, and tissue homeostasis in the kidney. *EMBO J.* **37**, e98615 (2018). 10.15252/embj.201798615 PMC6068446 29925518

[59] Ramachandran H, Herfurth K, Grosschedl R, Schafer T, Walz G (2015). SUMOylation Blocks the Ubiquitin-Mediated Degradation of the Nephronophthisis Gene Product Glis2/NPHP7. PloS One.

[60] Ganner A (2020). The acetyltransferase p300 regulates NRF2 stability and localization. Biochem. Biophys. Res. Commun..

[61] Thoma CR (2009). VHL loss causes spindle misorientation and chromosome instability. Nat. Cell Biol..

[62] Thoma CR (2007). pVHL and GSK3beta are components of a primary cilium-maintenance signalling network. Nat. Cell Biol..

[63] Simons M (2005). Inversin, the gene product mutated in nephronophthisis type II, functions as a molecular switch between Wnt signaling pathways. Nat. Genet..

[64] Wiznerowicz M, Trono D (2003). Conditional suppression of cellular genes: Lentivirus vector-mediated drug-inducible RNA interference. J. Virol..

[65] Kamath RS, Martinez-Campos M, Zipperlen P, Fraser AG, Ahringer J (2001). Effectiveness of specific RNA-mediated interference through ingested double-stranded RNA in *Caenorhabditis elegans*. Genome Biol..

[66] Aspernig, H.*, et al.* Mitochondrial Perturbations Couple mTORC2 to Autophagy in *C. elegans*. *Cell Rep.* **29**, 1399–1409 e1395 (2019). 10.1016/j.celrep.2019.09.072 31693882

