# Supplementary material for: VHL suppresses RAPTOR and inhibits mTORC1 signaling in clear cell renal cell carcinoma
Source: Sci Rep. 2021 Jul 21;11:14827. doi: 10.1038/s41598-021-94132-5 (PMC8295262; doi:10.1038/s41598-021-94132-5)

VHL suppresses RAPTOR and inhibits mTORC1 signaling in clear cell renal cell carcinoma

Athina Ganner[1], Christina Gehrke[1], Marinella Klein[1], Lena Thegtmeier[1], Tanja Matulenski[1], Laura Wingendorf[1], Lu Wang[1], Felicitas Pilz[1], Lars Greidl[1], Lisa Meid[1], Fruzsina Kotsis[1], Gerd Walz[1], Ian J. Frew[2], and Elke Neumann-Haefelin[1]*

Supplementary Figure 1: VHL interacting proteins and model of VHL30 and 19

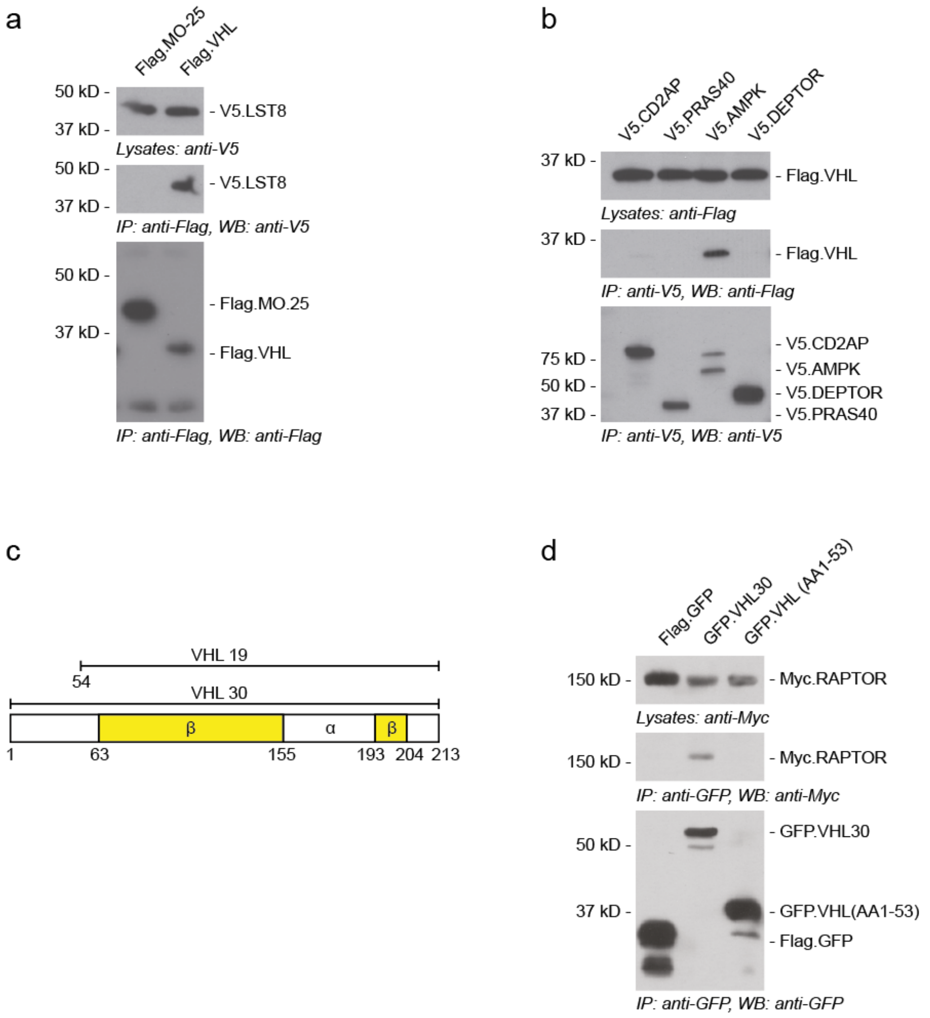

a) VHL interacts with LST8. Flag.VHL and V5.LST8 were transiently co-expressed in HEK293T cells. Flag.MO-25 was used as control. After immunoprecipitation (IP) with anti-Flag antibody, the immobilized LST8 was detected by Western blot (WB) analysis using anti-V5 antibody in the precipitate containing VHL, but not control protein (middle panel). Full-length blots are presented in Supplementary Figure 4.

b) VHL binds AMPK. Tagged proteins were transiently co-expressed as indicated in HEK293T cells. After immunoprecipitation (IP) with anti-V5 antibody, the immobilized VHL was detected by Western blot (WB) analysis using anti-Flag antibody in the precipitate containing AMPK, but not control protein, PRAS40 or DEPTOR (middle panel). Full-length blots are presented in Supplementary Figure 4.

c) Schematic representation of VHL isoforms with highlighted protein domains. The VHL protein contains 213 amino acid residues with a molecular weight of ~30 kD (VHL30). Internal initiation of translation at methionine 54 gives rise to a second VHL isoform with approximately 19 kD (VHL19). VHL has two domains: an N-terminal β domain containing a putative macromolecular binding site as well as a smaller C-terminal α domain which interacts directly with Elongin C.

d) VHL30 but not VHL(AA1-53) interacts with RAPTOR. Myc.RAPTOR and Flag.VHL30 or Flag.VHL (AA1-53) were transiently co-expressed in HEK293T cells. Flag.GFP was used as control. After immunoprecipitation (IP) with anti-Flag antibody, the immobilized RAPTOR was detected by Western blot (WB) analysis using anti-Myc antibody in the precipitate containing VHL30, but not VHL(AA1-53) (middle panel). Full-length blots are presented in Supplementary Figure 4.

Supplementary Figure 2: Interaction of VHL type 2B mutants with RAPTOR

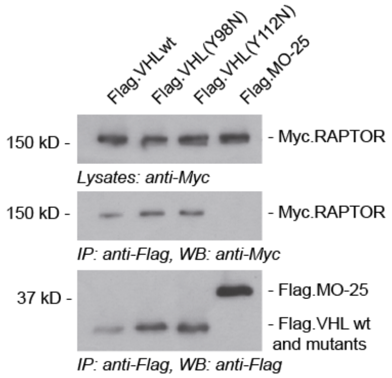

Wild type (wt) VHL, VHL(Y98N) and VHL(Y112N) were transiently co-expressed with RAPTOR in HEK293T cells. MO-25 was used as negative control. After immunoprecipitation (IP) with anti-Flag antibody, the immobilized RAPTOR was detected by Western blot (WB) analysis using anti-myc antibody in the precipitate containing wild type VHL as well as VHL type B mutants, but not in the lysate containing control protein. Full-length blots are presented in Supplementary Figure 4.

Supplementary Figure 3: RAPTOR levels are independent of HIF inhibition

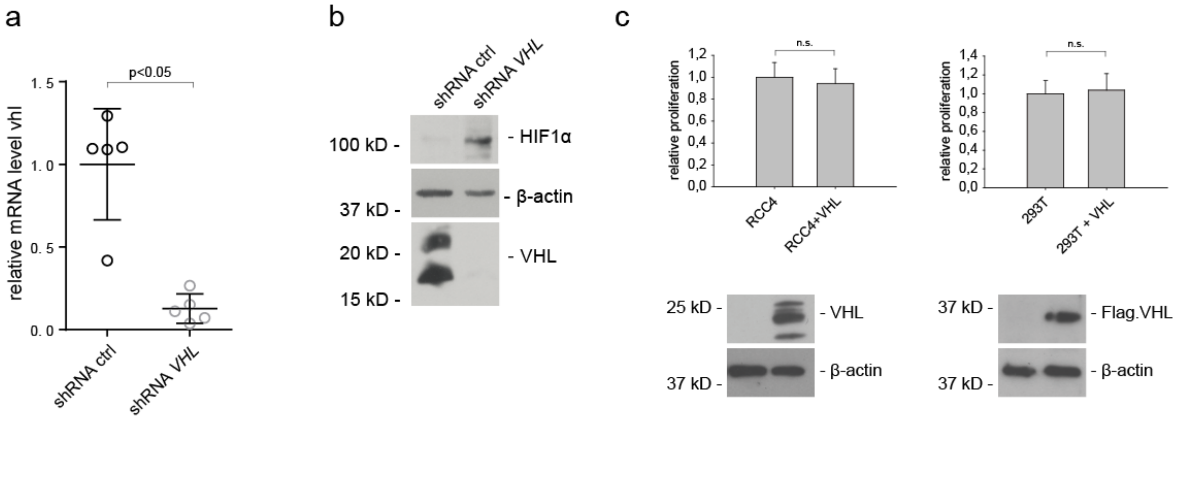

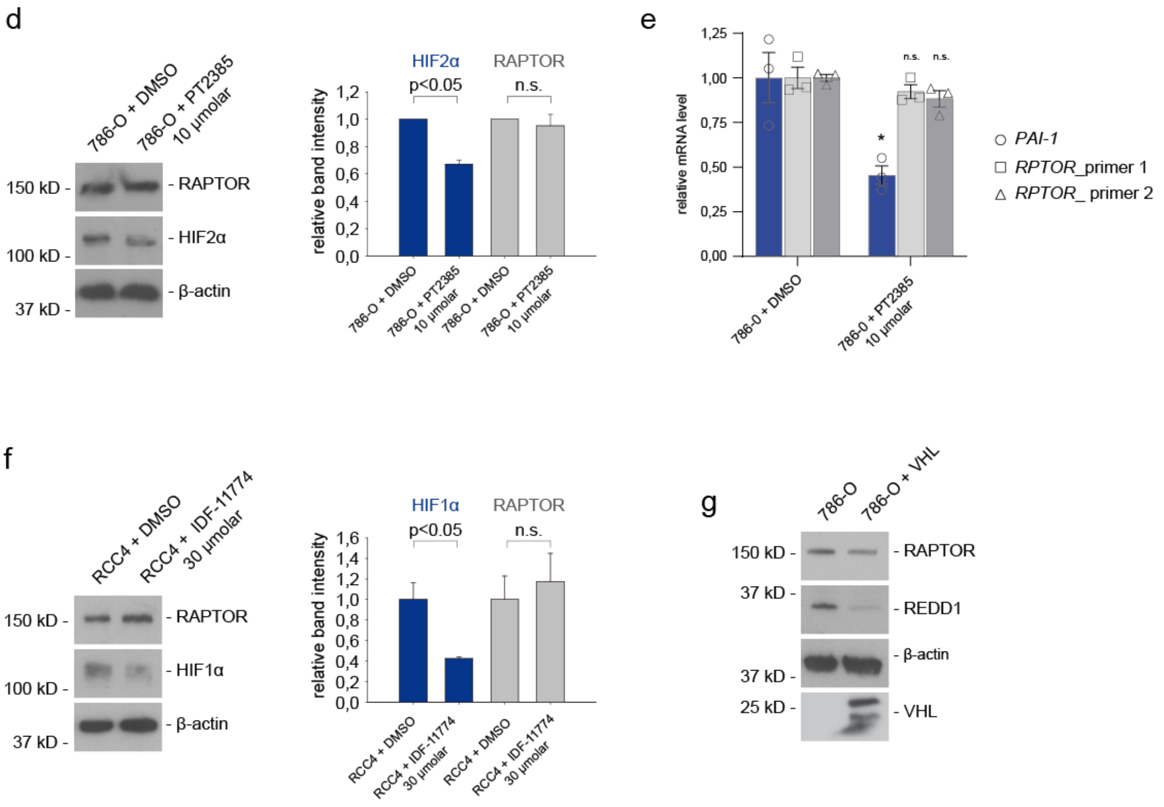

a) mRNA levels of *VHL* in HeLa cells infected with inducible control-shRNA or *VHL*-specific shRNA assayed by qPCR. n=5. Data are presented as mean ± SEM. $p < 0.05$ (t-test).

b) HIF1α is upregulated in *VHL*-deficient cells. Blot showing HIF1α expression in HeLa cells infected with lenti-control shRNA or lenti-*VHL* shRNA.

c) Cell proliferation is not affected by VHL. Cell viability of RCC4 cells re-expressing VHL and HEK293T cells transiently transfected with VHL was assayed compared to control. Expression of VHL was verified by immunoblotting analysis. Data are presented as mean ± SEM of 3 independent experiments. n.s., not significant (t-test).

d) RAPTOR protein levels are independent of HIF2α inhibition. 786-O cells were incubated with the HIF2α inhibitor PT2385 for 72 h and lysates were assessed by immunoblotting with anti-RAPTOR and anti-HIF2α antibody. Quantification of three independent experiments. Mean values ± SEM. n.s., not significant (t-test). Full-length blots are presented in Supplementary Figure 4.

e) *RPTOR* mRNA levels are independent of HIF2α inhibition. 786-O cells were incubated with the HIF2α inhibitor, PT2385, for 72 h at concentrations indicated. mRNA expression of *RPTOR* and *PAI-1* were assessed by q-PCR relative to HSPCB. Data are represented as mean ± SEM. *$p < 0.05$, n.s., not significant (t-test).

f) RAPTOR protein levels are independent of HIF1α inhibition. RCC4 cells were treated with the HIF1 α inhibitor IDF-11774 for 24 hours and cell lysates were analyzed for RAPTOR protein levels by immunoblotting. Data are represented as mean ± SEM. *$p < 0.05$, n.s., not significant (t-test).

g) VHL suppresses RAPTOR in a REDD-1-independent manner. Lysates of 786-O cells and 786-O cells expressing VHL were analyzed by immunoblotting for RAPTOR and REDD1 expression levels.

Supplementary Figure 4: The original Western blots from Figures 1–5 and Supplementary Figures 1-3

Figure 1a

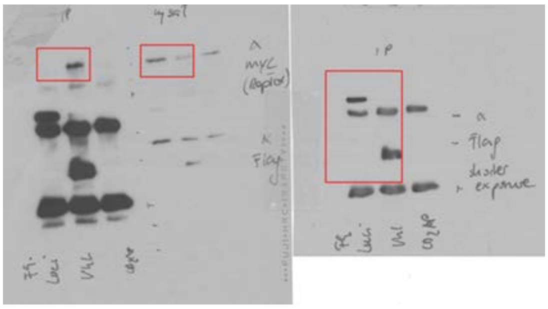

Figure 1e

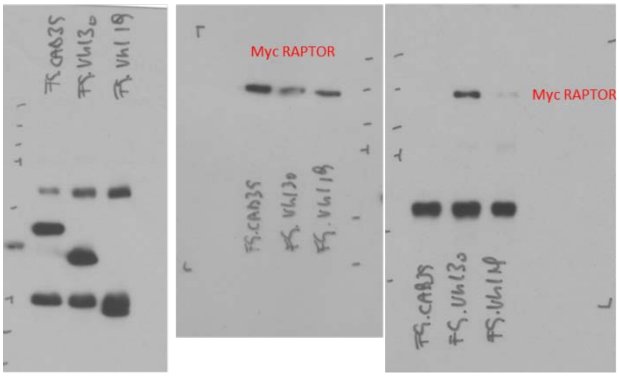

Figure 1b

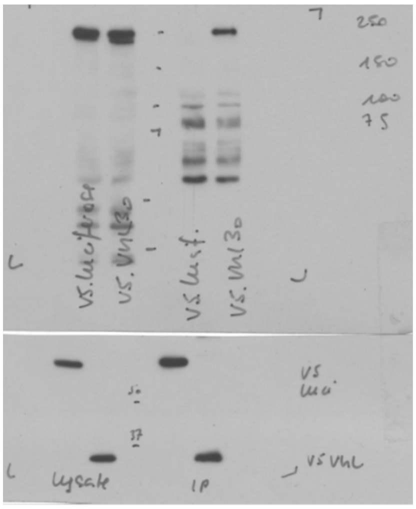

Figure 1f

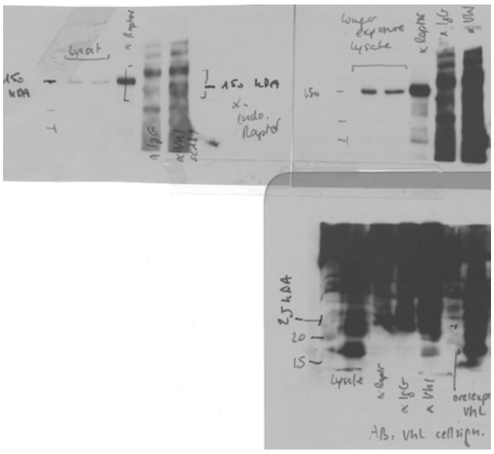

Fig. 1c

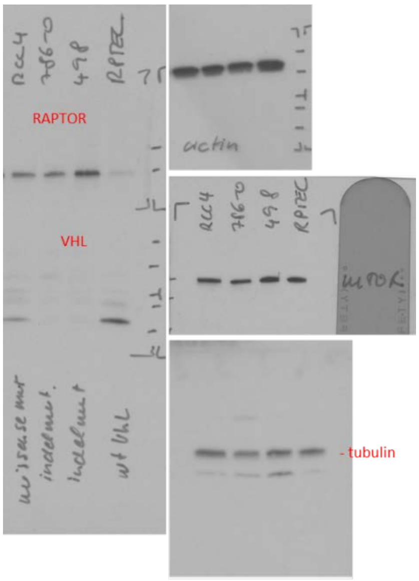

Figure 2a

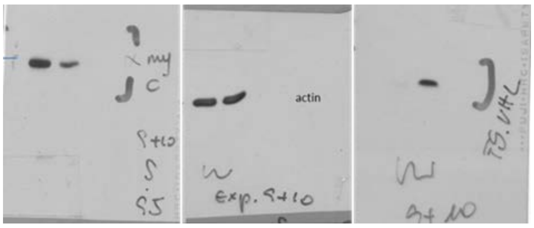

Figure 2b

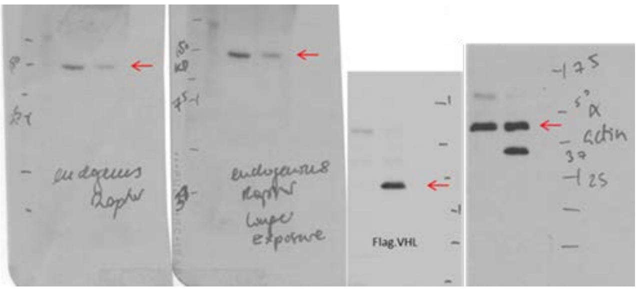

Figure 2c

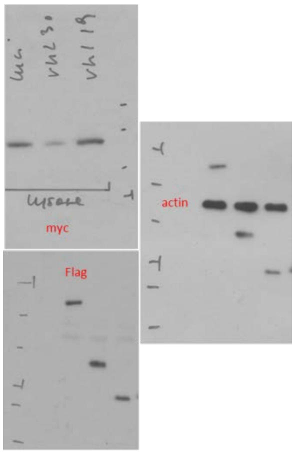

Figure 2d

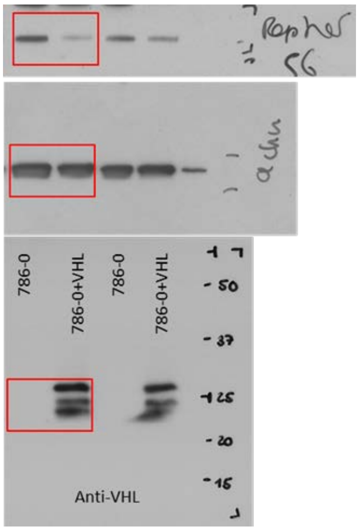

Figure 2e (left)

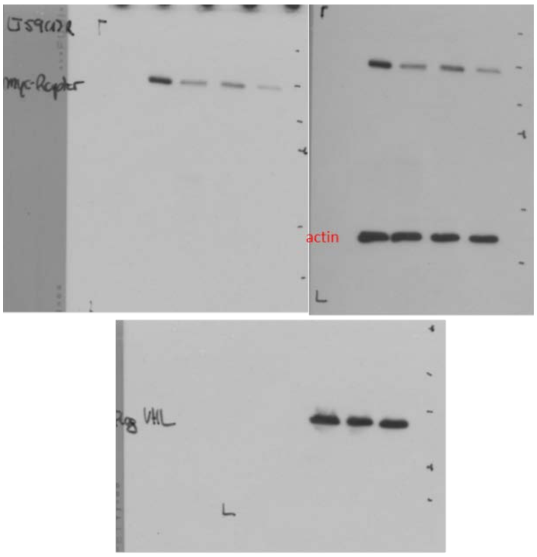

Figure 2e (right)

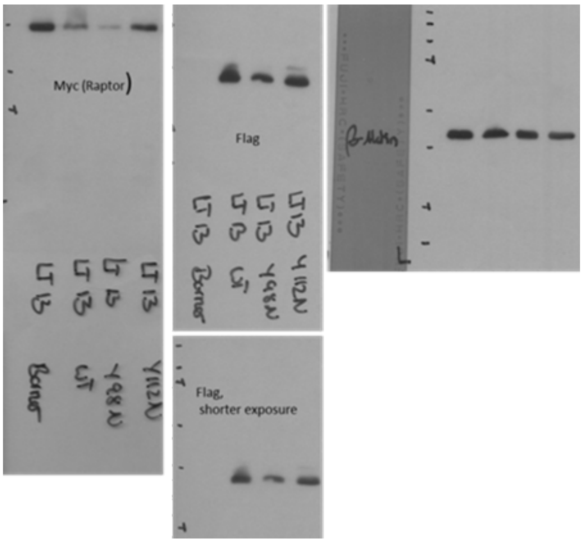

Figure 3a

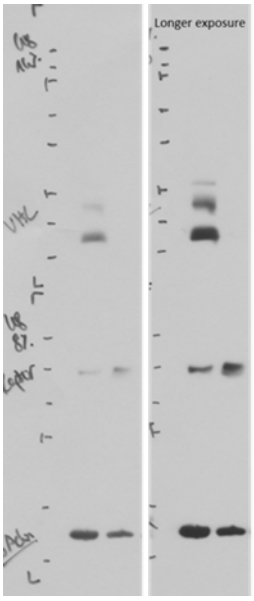

Figure 3d

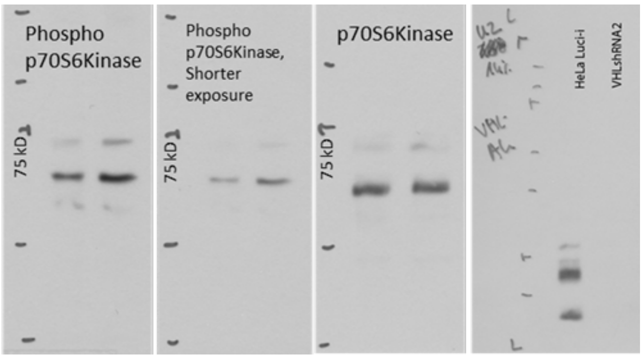

Figure 3e

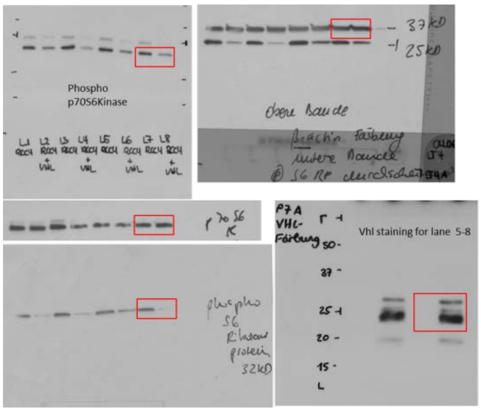

Figure 4a

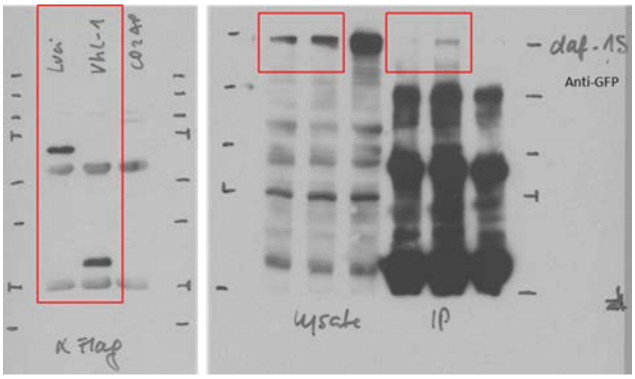

Figure 3f

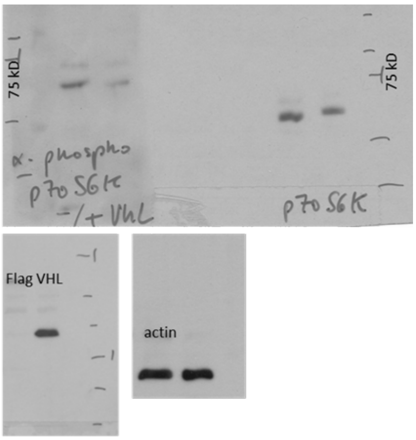

Figure 4b

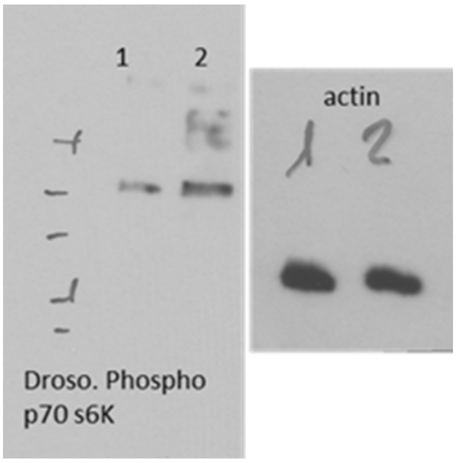

Figure 3g

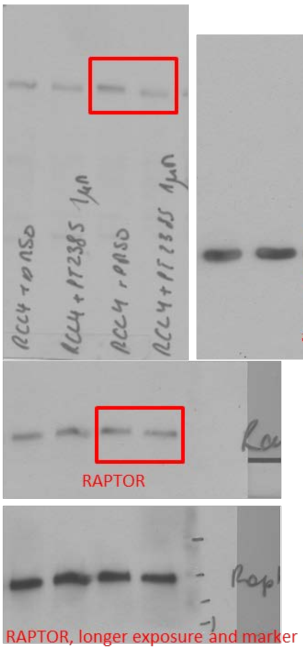

Figure 5a

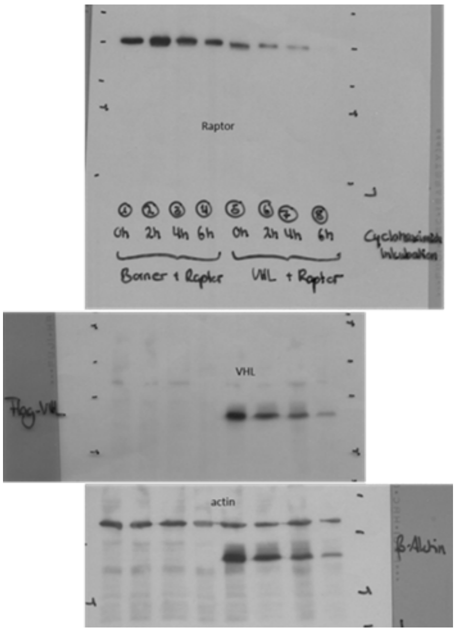

Figure 5b

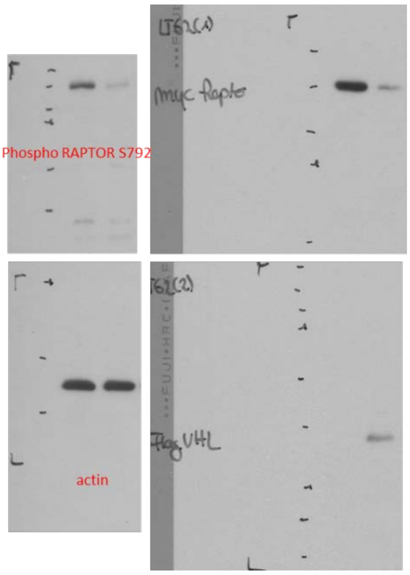

Supplementary Figure 1a

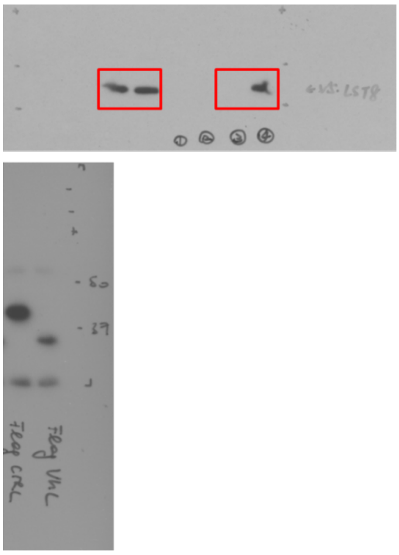

Figure 5c

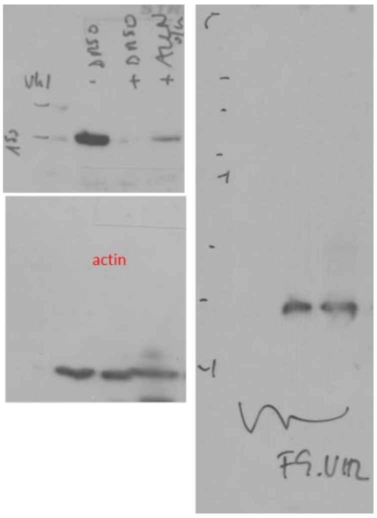

Supplementary Figure 1b

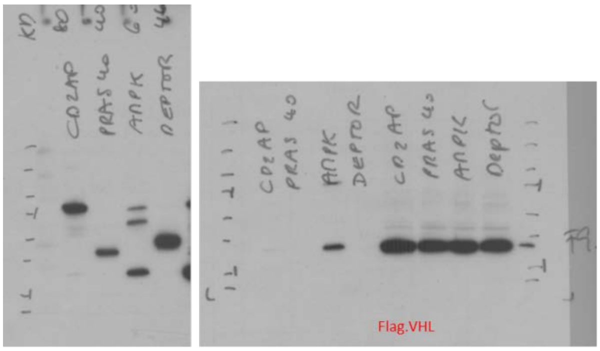

Supplementary Figure 1d

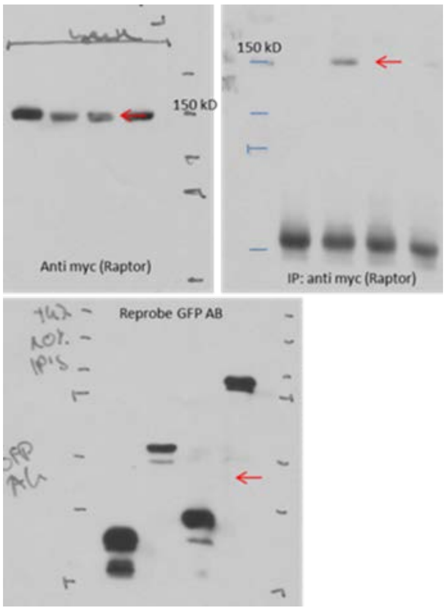

Figure 5d

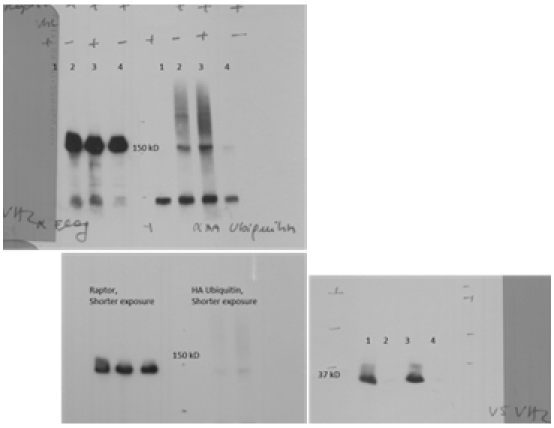

Supplementary Figure 2

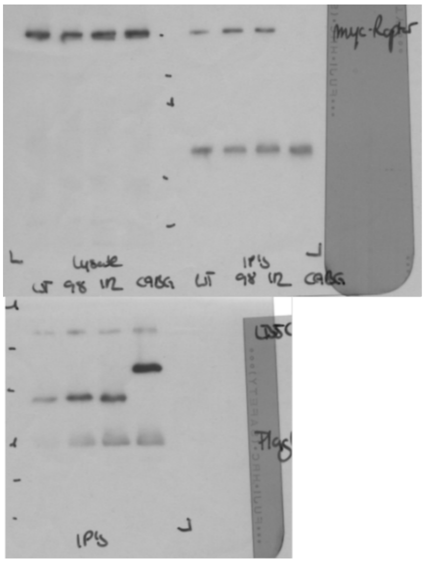

Supplementary Figure 3d

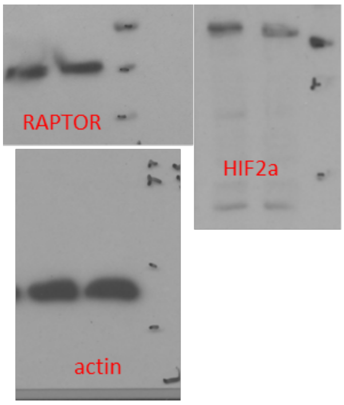

Supplementary Figure 3f

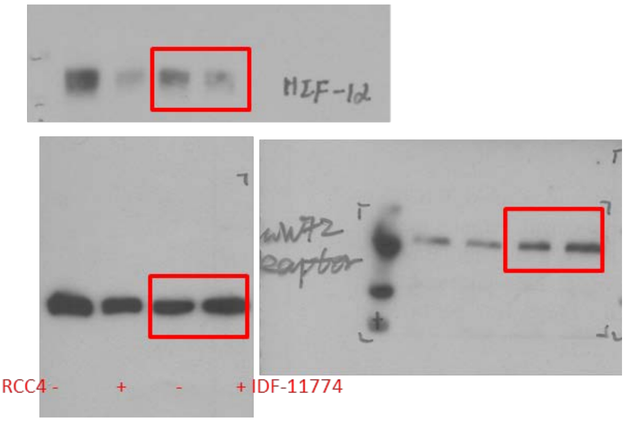

Supplementary Figure 3b

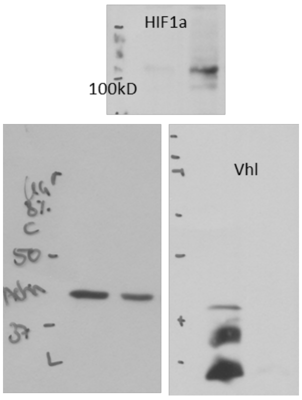

Supplementary Figure 3g

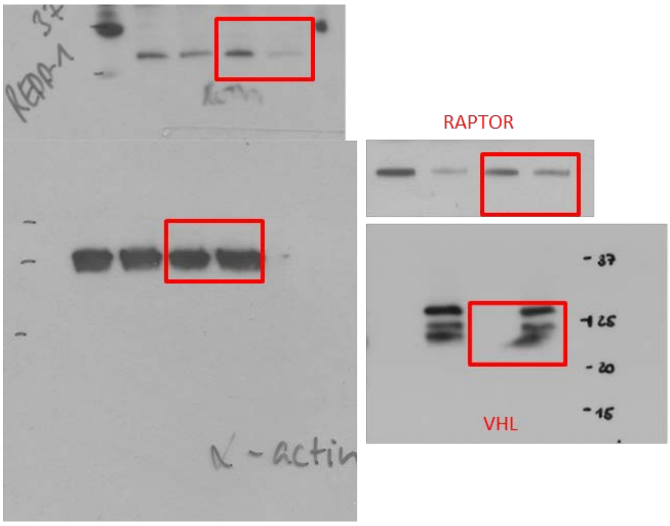

Supplementary Figure 3c

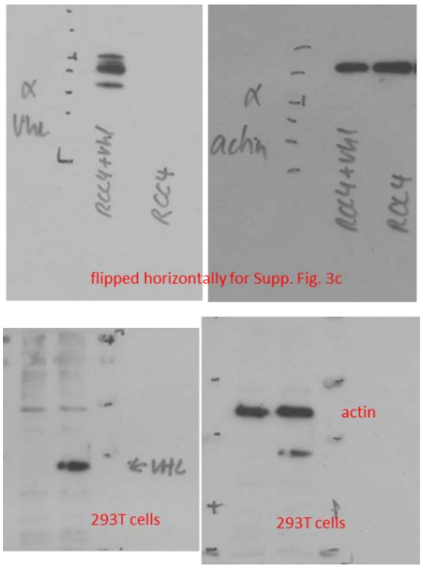

Supplement: Supplementary file 1 — Supplementary Information. [file 41598_2021_94132_MOESM1_ESM.pdf]
